# Mapinsights: deep exploration of quality issues and error profiles in high-throughput sequence data

**DOI:** 10.1093/nar/gkad539

**Published:** 2023-06-28

**Authors:** Subrata Das, Nidhan K Biswas, Analabha Basu

**Affiliations:** National Institute of Biomedical Genomics, Kalyani, 741251, West Bengal, India; National Institute of Biomedical Genomics, Kalyani, 741251, West Bengal, India; National Institute of Biomedical Genomics, Kalyani, 741251, West Bengal, India

## Abstract

High-throughput sequencing (HTS) has revolutionized science by enabling super-fast detection of genomic variants at base-pair resolution. Consequently, it poses the challenging problem of identification of technical artifacts, i.e. hidden non-random error patterns. Understanding the properties of sequencing artifacts holds the key in separating true variants from false positives. Here, we develop Mapinsights, a toolkit that performs quality control (QC) analysis of sequence alignment files, capable of detecting outliers based on sequencing artifacts of HTS data at a deeper resolution compared with existing methods. Mapinsights performs a cluster analysis based on novel and existing QC features derived from the sequence alignment for outlier detection. We applied Mapinsights on community standard open-source datasets and identified various quality issues including technical errors related to sequencing cycles, sequencing chemistry, sequencing libraries and across various orthogonal sequencing platforms. Mapinsights also enables identification of anomalies related to sequencing depth. A logistic regression-based model built on the features of Mapinsights shows high accuracy in detecting ‘low-confidence’ variant sites. Quantitative estimates and probabilistic arguments provided by Mapinsights can be utilized in identifying errors, bias and outlier samples, and also aid in improving the authenticity of variant calls.

## Graphical Abstract

**Graphical Abstract:**
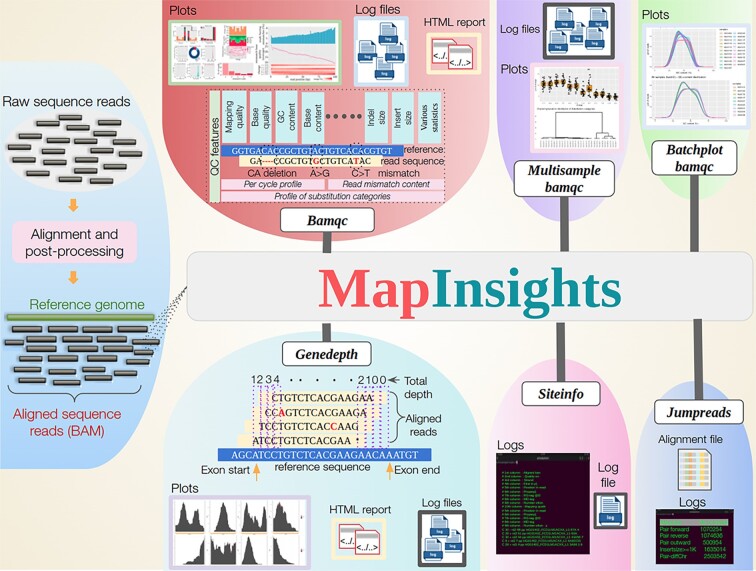

## INTRODUCTION

High-throughput sequencing (HTS) technologies facilitate deep exploration of genomes of various organisms and help in understanding the complexities of biological processes (1). Over the last decade, the field of HTS has gone through multiple trials with sequencing chemistry and seen tremendous improvements in terms of quality and data yield, with reducing cost and turnaround time (1–3). This has resulted in HTS gaining applicability in a wider field beyond biomedical sciences, ranging from agricultural to veterinary research, microbiome to viral genome, and human evolution to disease diagnosis and treatment (4–12). Although HTS typically has low error rates, because it generates an enormous amount of data in every run (ranging from a few hundred to >10 000 Gb), the overall error burden is high. Left unidentified, these errors can be extremely costly as they can creep into decision-making processes in human diseases as well as other aspects of biomedical sciences. It is therefore crucial to perform intense quality control (QC) to remove technical artifacts, which are typically non-random and can lead to erroneous conclusions (13,14).

HTS laboratory protocols are complex (15,16). Technical artifacts are initiated and introduced at different stages during data generation and analysis. These artifacts can broadly be classified into three categories: (i) pre-sequencing error; (ii) sequencing error; and (iii) data processing error (17,18). The major source of pre-sequencing errors takes place during library preparation, e.g. artificial C:G to A:T transversion induced during DNA fragmentation because bases get oxidized by energy produced through ultrasonic shearing (19); C:G to T:A transition errors generated by spontaneous deamination during the polymerase chain reaction (PCR) process (20); and 8‐oxo‐G errors occur due to heat, shearing and metal contaminants (C:G to A:T) (21,22). Sequencing errors can also arise due to overlapping/polyclonal cluster formation and optical imperfections (13,23). Erroneous end-repair and accumulation of phasing and pre-phasing problems in sequencing lead to the elevated error rate at the end of the reads (13,14). Lastly, bioinformatics tools such as mapping algorithms can generate errors due to the limitations of the method or erroneous coding along with the inaccuracy or incompleteness of the reference genome (18). While these are a few artifact-inducing processes, there exist some other intricate aspects such as genomic depth of coverage (DCOV) or inefficiency of variant callers that can substantially affect experimental results and data interpretation. In targeted sequencing, DCOV suffers from insufficiency and inconsistency even when the sequencing is performed at 120× (24). Non-uniformity in depth is primarily attributed to GC content that introduces bias during DNA amplification by PCR and inherent properties of the genome such as repeat elements, segmental duplications as well as capture efficiency of design probes (25–27). Additionally, due to incompleteness of variant detection tools, variant callers reject true sites or accept artifacts primarily due to the use of pre-defined fixed filters or programming limitations.

A few past studies have investigated error profiles which are specific to Illumina sequencers and have disclosed various error characteristics. (i) Errors are not completely random, but rather are probably associated with sequence motifs (GGC, GGT and GTG/AGC) mainly preceded by base G resulting in artifacts along with inverted repeats (13,17,28–32). (ii) Error rates differ by nucleotide substitution types; some studies show that A>C transversions are the most frequent error (17,29,31). (iii) Base quality and errors have bias towards read position, length and read orientation (29,32–34). (iv) Differences in error rate as observed in recent Illumina instruments (34–36). However, such error profiles are unknown for other orthogonal sequencing technologies such as element biosciences, MGI, etc., since these platforms are relatively new and the sequencing data generated by these platforms have not been explored comprehensively by the community. Further, several studies measured biases with respect to read alignment in different parts of the genome essentially by calculating depth bias (37–39).

All of these systematic errors and biases make HTS data analysis complex and difficult, especially when it comes to identification of authentic variants and separating them from ‘false-positive’ variant calls. There have been concerns about next-generation sequencing (NGS) data quality with respect to artifacts in variant calling; some analytical strategies (40,41) have been proposed that focus on detecting and flagging problematic regions in the genome for variant calling. Although recent variant callers used deep learning methods to deal with sequencing errors, their accuracy depends on training data. Effective training of such variant callers required ground truth knowledge of various error patterns and biases pertaining to different sequencing technologies, chemistries and library protocols where sequence data QC tools could play an important role, in particular those that report various metrics related to technical artifacts and biases. Few bioinformatics methods exist to identify some types of technical errors and biases from sequencing data and detect outliers based on batch analysis of QC results. Tools such as Qualimap2 (42), Picard (https://broadinstitute.github.io/Picard/), Samtools-stats (43), FastQC (http://www.bioinformatics.babraham.ac.uk/projects/fastqc/) and Alfred (44) were developed to address some of these technical hurdles related to sequence data quality. Normally these tools report QC metrics such as mapping quality, insert size, nucleotide content, GC content, base quality and error rate. The above tools, except Picard which additionally calculates oxo-G errors and bait bias, provide an overall error rate. While Qualimap2 and Alfred perform multisample analysis on their own QC metrics, another popular tool MultiQC (45) facilitates batch analysis of QC reports generated by other QC tools. None of the tools focuses on base mismatches with respect to reference nucleotides, nor do they enable multisample analysis based on base mismatches with respect to nucleotides present in the reference genome. Further, several tools, e.g. SeqControl (46) and CovReport (47), perform QC analysis based on depth profile. SeqControl is primarily developed for whole-genome/exome segments, whereas CovReport can estimate depth on the gene level. These methods are oblivious of variability and other essential and pertinent aspects (GC content, coverage at different depths) with respect to gene depth, specially at the exon level.

Here, we present Mapinsights, a toolkit that aims to facilitate detailed QC analysis of aligned sequence data. Six modules were developed as part of the toolkit. Each module has its own advantages and importance with respect to detection of various quality issues and technical artifacts. We have used both open-source data and new datasets to demonstrate the usefulness of our tool and subsequently identify potential errors and biases present in both whole-genome and exome sequencing data that were generated as part of international genome projects. The tool also identifies outlier samples with respect to sequencing errors, detects biases across various sequencing platforms and identifies issues in popular Illumina-NovaSeq sequencing data. As technical artifacts are known to be the key confounding factors associated with sensitive detection of subclonal mutation, assessing the accuracy of such variants with respect to the error profile is important for research outcome, disease diagnosis and treatment follow-up. Specific features of our tool generate a quantitative estimate that is capable of identifying low confident variant sites with high accuracy. One cardinal feature which separates Mapinsights from most available data QC software is its comprehensive analysis of each individual sequence; a ‘personalized QC package’ in the age of ‘personalized genomics’.

## MATERIALS AND METHODS

### Algorithm

**Figure 1.**
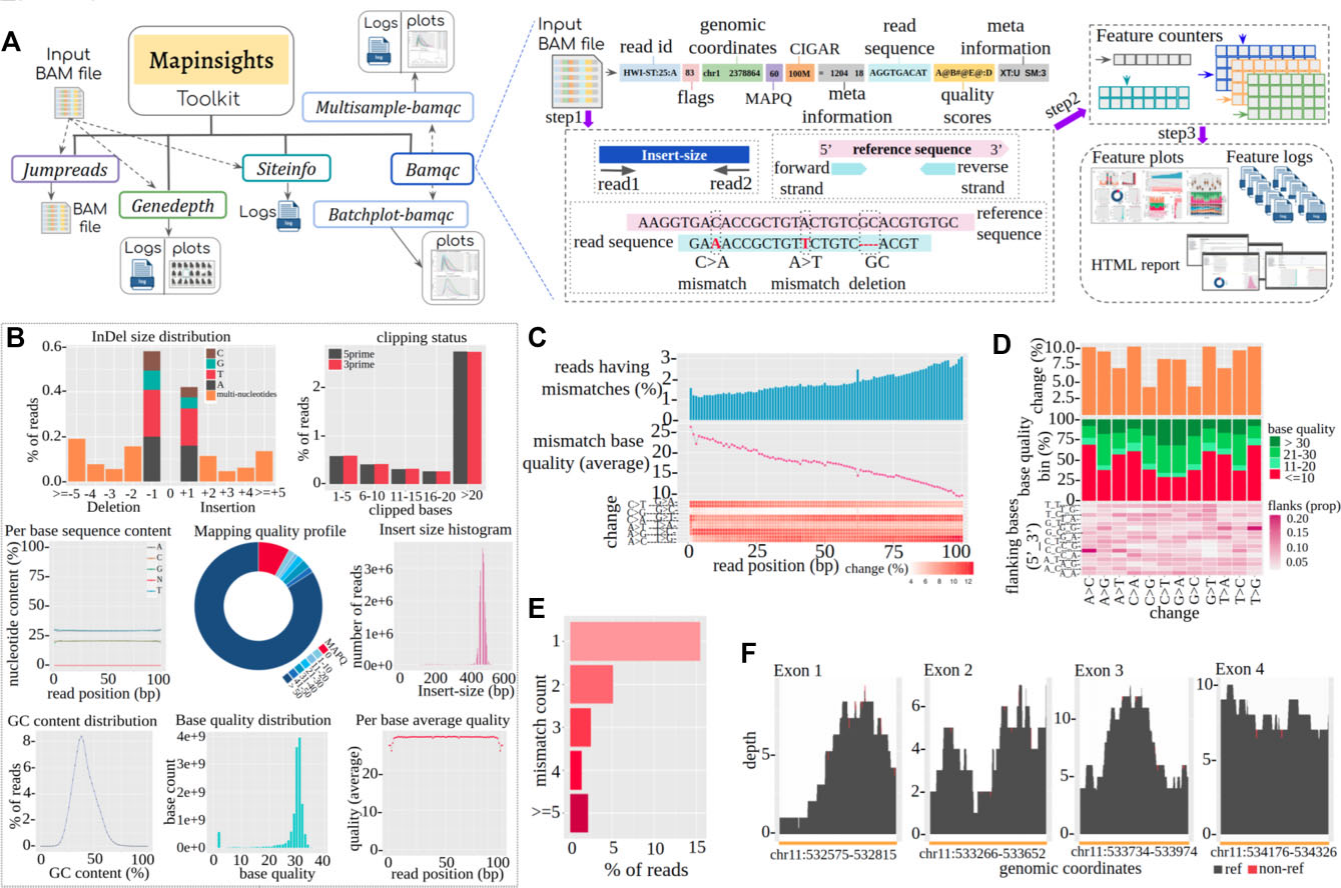
(**A**) General overview of the Mapinsights toolkit with overall workflow of the *bamqc* module. (**B**) (From left to right) Length distribution of InDels: it is observed that 0.57% and 0.42% of reads have single base deletions and insertions, respectively. Length distribution of clipped bases: 2.7% of reads have been clipped (from 5′ and 3′ ends), length ≥ 20 bases. Per base sequence content reports that the proportions of A, T and G, C bases are ∼29% and ∼21%, respectively. Mapping quality profile and insert size distribution: the insert size distribution has very little variance, with a mean insert size of 454.9 bp, and >80% of reads have mapping quality > 50. GC content distribution: among all reads, >95% of reads have a GC content ranging from 20% to 62%. Base quality distribution: most of the bases (∼94%) have quality value ≥ 20. Per base average base quality: 92% of read positions have average base quality ≥ 28. (**C**) *Per-cycle-profile*: per cycle substitution rate, i.e. the percentage of reads having mismatches with respect to the reference genome on a per cycle basis (upper panel), average quality of mismatched bases per cycle (middle panel) and percentage occurrence of different substitution categories per cycle (lower panel). We observe (i) a monotonic trend of a decrease in quality with the number of cycles and (ii) a strong correspondence of the increased mismatch at certain sites where the average quality is also depleted. **(D**) *Profile-of-substitution-categories*: distribution of substitution categories (upper panel), quality values of mismatched bases per substitution category in a bin-wise fashion (middle panel) and the lower panel depicts the distribution of flanking bases (5′–3′) per substitutional category. (**E**) *Read-mismatch-content*. (**F**) Exon-wise coverage plots of a specific gene (*HRAS*).

The Mapinsights algorithm was designed to be easily integrated into an existing NGS data analysis pipeline. Six analytical modules were developed within the Mapinsights toolkit for post-alignment QC analysis of alignment files: namely *bamqc*, *multisample-bamqc*, *batchplot-bamqc*, *siteinfo*, *genedepth* and *jumpreads*. A graphical overview of the toolkit is shown in Figure 1A. The functionality and importance of QC features and modules of Mapinsights are summarized in Table 1. A comparative overview of different QC tools that are widely used by the community can be found in Supplementary Table S1. A coordinate sorted binary alignment map (BAM) file generated from alignment of paired-end reads is the primary input to Mapinsights; however, other parameters and input files can be fed in based on the type of analysis intended. All backend code is open source, written in C and R packages. During code development, samtools libraries were used extensively.

**Table 1.** Summary of various novel features and modules of Mapinsights

Mapinsights modules	Features	Functionality and importance
BAMQC		
	*Per-cycle-profile*	False variant sites are often introduced by cycle bias. This module provides the assessment of substitutional^a^ cycle bias present in the align data. *Per-cycle-profile* facilitates the detection of cycle-specific biases in the datasets. This feature also reflects per cycle base quality bias and intensity of each substitution category per cycle. It helps to identify read position-specific biases and aid detection of artifactual variant sites.
	*Profile-of-substitution-categories*	This module provides category-wise substitution percentages that capture possible bias in the data. For example, excess C > A/G > T and C > T/G > A changes are observed in oxo-G and FFPE-specific errors, respectively. An overall idea about the quantitative contribution of such errors can be obtained from this feature. Additionally, substitution category-wise base quality and flanking base distribution enable the detection of known and new sequencing biases. The information provided by this feature can be used as a prior during variant filtering and refinement.
	*Read-mismatch-content*	This specific feature provides the detailed assessment of data quality in terms of percentage of reads having mismatch with respect to the reference genome. A quantitative assessment of error variation across different lanes/runs can be obtained from this feature or even from read pairs (read1 versus read2). It also provides a means to cross-compare different alignment algorithms with respect to mapping performance against a reference sequence.
MULTISAMPLE-BAMQC		This module analyses output of the *bamqc* module in multisample mode and performs cluster analysis based on novel and existing features to evaluate consistency among samples and outlier detection.
BATCHPLOT-BAMQC		Given the *bamqc* results of two groups, this module enables side-by-side batch plots of the group for better comparison and helps in visually identifying any outliers.
GENEDEPTH		It is known that depth of coverage varies across genomic regions, and potential mutations can be missed or false-positive variants can be detected due to no or low depth. This module will provide a clear view about the uniformity of depth over the gene and reports if all exons are adequately covered with the expected depth of coverage for a given gene. Thus it provides a sense of whether there exists an issue with depth of coverage for the gene of interest. Even though the module is designed primarily for genes, it can easily be utilized for other targeted genomic regions as well.
SITEINFO		To minimize potential false positives, different variant callers use their own filtering criteria and cut-offs which are mostly pre-defined and fixed. This increases the chance to accept artifacts and reject true variants (true hotspot sites were known to be missed in low depth scenarios) by the variant caller due to the algorithmic limitations. This module provides a way to generate comprehensive information about specific variant sites of interest that help both detection of true variants and rejection of false ones.
JUMPREADS		This provides an estimation of atypical reads present in the data with respect to mapping properties that help in detection and validation of structural variants present in the data. This module also extracts relevant reads for downstream analysis.

^a^A substitution is defined as a sequence base mismatch in the sequenced read with respect to the reference base.

### bamqc

The *bamqc* module analyzes BAM files to calculate in-depth QC matrices primarily based on different mapping properties, base quality, nucleotide mismatches with respect to reference bases, etc., and presents them in the form of plots for easy understanding. This specific module takes both BAM files and reference genomes as input and parses the BAM file to generate advanced QC analysis based on the mapped reads. In step 1: the *bamqc* module extracts necessary information pertaining to a read from the relevant fields of the input BAM and stores it in respective pre-allocated data structures. In step 2: each sequence read is aligned back to the reference genome using the obtained information such as Compact Idiosyncratic Gapped Alignment Report (CIGAR), chromosomal number and coordinates. After realignment, pertinent counters allotted for different features are updated based on respective criteria. A detailed flowchart of the algorithm can be found in Supplementary Figure S1. Certain feature information is collected in a categorical fashion, i.e. in an overall and paired-end orientation (read1+, read1–, read2+, read2–) manner. Following the compilation of BAM parsing, a large number of QC matrices are calculated and written in different log files out of which a certain fraction of features are presented in the form of plots. *bamqc* facilitates the QC on the BAM file based on read groups. It can generate region-specific QC matrices by taking genomic regions in bed format. The results of the QC analysis are presented as a static report in HTML containing summary statistics and plots; however, all the log files are kept in a folder as a plain text file suitable for parsing and further processing.

Along with common QC features, the *bamqc* module also includes novel features such as *per-cycle-profile* comprising three subfeatures: (i) proportion of reads having mismatches with respect to the reference nucleotide on a per cycle basis; (ii) average quality of mismatched bases per cycle; and (iii) proportion of occurrences of different substitution categories per cycle. *Profile-of-substitution-categories* includes three subfeatures: (i) distribution of substitution categories; (ii) quality values of mismatched bases per substitutions category; and (iii) distribution of flanking bases (5′–3′) per substitutional category. Lastly, *read-mismatch-content* generates metrics on the proportion of reads with numbers of mismatches.

The *bamqc* module reports an extensive number of QC metrics and plots for easy visualization. The module outputs various QC metrics such as number of mapped reads, number of unmapped reads, number of QC-failed reads, number of secondary reads, number of supplementary reads, adapter content, nucleotide content, strand ratio, mean read length, mean DCOV, error rates and many other relevant QC logs. A few common QC features such as distribution of base quality, mapping quality, per base nucleotide content, average quality, insert size and GC content are presented as plots. These metrics are capable of detecting a few of the sequencing issues as other tools do.

In addition, a number of new features (based on base mismatches with respect to nucleotide in the reference genome) have been included in the module for easier identification of known and new sequencing issues (errors and biases). Features such as *per-cycle-profile* included in the *bamqc* module are capable of detecting cycle bias [when an elevated number of mismatches is observed in one or few cycle(s) compared with its adjacent cycles] which is known to affect the variant calls. Whether any particular cycle(s) has any bias regarding base quality or any substitution type can also be detected from the same feature. In addition, the *profile-of-substitution-categories* feature enables identification of errors and biases with respect to particular types of substitution. Cases where any type of substitution has a preponderance or quality issues or occurs abundantly in certain flanking bases compared with other types (which reflect biases) can easily be identified from the plots and logs. Further, the feature *read-mismatch-content* is able to identify mismatch variation across different sequencing lanes/runs showing the proportion of reads containing the exact numbers of mismatches. It also can be used to cross-compare different alignment algorithms and quantify the differences. See Table 1 for more details.

Another key advancement of the *bamqc* module is that all these new features are also reported in a paired-end orientation manner which can potentially identify whether the observed errors or biases have any read (first-in-pair/second-in-pair) or strand (forward/reverse) specificity. Sequencing errors and biases can effectively address if the read and strand level specificity are known.

Two more modules are developed in relation to the *bamqc* module, namely *multisample-bamqc* and *batchplot-bamqc*. Analyzed results obtained using the *bamqc* module from multiple samples are the primary input of the modules. *multisample-bamqc* facilitates comparison analysis across multiple samples that allow evaluation of consistency among samples and outlier detection. This module performs hierarchical cluster analysis to estimate the variability between analyzed datasets based on novel features (*per-cycle-profile*, *profile-of-substitution-categories*, *read-mismatch-content*) as well as a few other specific features such as mapping quality, insert size and GC content distribution. First the *multisample-bamqc* module prepares an M × N matrix where M represents samples and N represents possible feature values, say for example the insert size feature is a cell element (*e_ij_*) of the matrix representing the proportion of reads in sample *i* with an insert size of *j*. The Euclidean method is used to calculate the distance followed by clustering using Ward's method with default parameters. Further, given the *bamqc* results of two groups, *batchplot-bamqc* enables side-by-side batch plots of the group for better comparison and helps in identifying any outliers.

### genedepth

The *genedepth* module calculates exon-wise DCOV and represents it as a graph for a given gene along with other relevant information such as exon length and GC content. The gene (exon-wise) bed file along with BAM and the reference file are the main inputs to this module. It starts by parsing the bed file. For each bed entry, the tool extracts only those reads that overlap with the bed interval and organize them in a pileup format. The pileup is then scanned and counts the number of reference and non-reference bases at each locus which add up to the total depth. A detailed flowchart can be found in Supplementary Figure S2A. DCOV plots together with a few statistics are calculated based on the information acquired and stored in log files. Moreover, all the results are presented as a static HTML report. The *genedepth* module is capable of identifying depth-related biases in exon-level resolutions. See Table 1 for more details.

### siteinfo

The *siteinfo* module queries the genomic locus in BAM files and provides comprehensive information about the alignment in query sites such as which nucleotides are present in that site, their base quality, strands, read mapping quality, insert size and read group. The primary inputs to the *siteinfo* module are BAM, the reference genome and chromosomal coordinate(s). Depending on query locations, the module extracts all the reads aligned to that particular coordinate and arranges them in pileup fashion. All necessary information corresponding to each extracted read is stored in appropriate data structures. An alignment index file is used for faster read extraction for pileup formation. *siteinfo* facilitates both single and batch query; it takes a file containing a list of coordinates as input or a single query coordinate directly from the command line as a parameter. A detailed flowchart can be found in Supplementary Figure S2B. Extensive details of mapping, base quality and meta information such as the read group about the genomic locus facilitated by the *siteinfo* module can be utilized for QC of a variant site. See Table 1 for more details.

### jumpreads

The *jumpreads* module is designed to extract reads with atypical alignment properties such as a long distance between the alignment of read pairs and exceptions in orientation. *jumpreads* acts on a coordinate sorted BAM file which is the prime input of the module. It begins by scanning the alignments and extracted reads based on mapping properties such as extra long insert, read paired mapped to different chromosomes and alignment orientation. For each read, the flag field together with insert size and mapping contig are examined in order to select the intended reads. As an output of the module, all selected reads are stored in a BAM file. A detailed flowchart can be found in Supplementary Figure S2C. The *jumpreads* module can give an estimation of atypical reads (reads with unusual mapping characteristics generally used for structural event detection) present in the alignment file (see Table 1).

Further, Mapinsights can easily be installed and applied on mapped high-throughput sequence data. The present version of the tool is not designed for single-end data which is one limitation of the tool. Mapinsights is primarily developed to perform quality control analysis on paired-end sequence data only.

### Accuracy testing

In order to test the accuracy of the tool, we have used simulated data. The wgsim module embedded within samtools (43) was used to generate 1 million paired-end reads from the hg19 decoy reference genome with default parameters. The reads are then aligned using BWA mem (48) and sorted by samtools. Summary statistics and different logs such as mapped read counts, forward read counts, number of bases, number of mismatches and InDel counts were calculated from sam-flag, MD tag and CIGAR string present in the BAM file, with the help of samtools and linux commands, which then compare with Mapinsights output. Further, a small fraction of mismatch sites and their flanking bases were checked through Integrative Genomics Viewer (IGV) (49). Additionally, two small BAM files were created, one comprising 100 insertion events of different length and the other containing 100 deletion events of different length, and the Mapinsights tool was run. All InDels and associated logs generated by the tool are then manually verified through IGV to assess the accuracy of the tool.

### Datasets

We have used open-source data, both whole-genome (WGS) and whole-exome (WES) datasets, from various sequencing platforms such as Illumina (HiSeq, NovaSeq), Element biosciences (AVITI), MGI (BGISEQ) and different consortium projects for this study. The following are the sequence data downloaded from various sources: (i) whole-genome data of NA12878 and HG01402 samples (50–55); (ii) 30 exome sequencing (HiSeq) alignment files from the 1000 Genomes project (50); (iii) two sets of exome sequencing data of NA12878, sequenced using HiSeq with the library prepared using two different library preparation kits, i.e Truseq and Nextera downloaded from the Genome-in-a-bottle (GIAB) project (52); and (iv) exome sequencing data of two lung adenocarcinoma patients comprising three types of data (a) fresh frozen normal tissue, (b) fresh frozen tumor tissue and (c) formalin-fixed paraffin-embedded (FFPE) tumor tissue (56). See Supplementary Table S2 for more details.

### Processing of datasets

High-throughput sequencing data in the FASTQ format were processed according to the GATK best practices protocol (57). BWA mem was used to align the sequenced reads against hg19 decoy reference sequences. Sorting and mark duplicates were performed by PICARD followed by InDel realignment and base quality recalibration using GATK (58). Data source (for details see Supplementary Table S2) and other data processing details can be found in the Supplementary methods.

### Speed and memory requirement

We have run the *bamqc* module of Mapinsights on one ∼15× WGS and one ∼30× WES alignment file comprising ∼324 million and ∼34 million reads, respectively. Mapinsights took ∼36 min to complete the QC process for the WGS file and ∼3 min for the WES file using Intel® Core™ i7-9700 CPU @ 3.00 GHz with 1 core and 32 GB of RAM. During the analysis process, runtime memory consumption was minimal (<500 MB). Run time summarization of different modules can be found in Supplementary Table S3. We also compare the runtime of Mapinsights and existing QC tools on a ∼15× NA12878 whole-genome sample. See the Supplementary methods for cross-tools comparison and other details.

## RESULTS

### Mapinsights toolkit: module and feature summary

To exemplify the basic functionalities of the different modules of Mapinsights, we used publicly available low coverage whole-genome data (HG01402) downloaded from the 1000 Genomes Consortium as a case study.

The *bamqc* module performs QC analysis of a paired-end alignment file that includes common features of the other tools along with new features such as *read-mismatch-content* profile, *profile-of-substitution-categories* (distribution of substitutions; quality and nucleotide context of mismatches) and *per-cycle-profile* [substitution rate, mismatch base quality (average) and the intensity of occurrence of substitution categories]. The *bamqc* module takes an alignment file along with a reference genome as input and generates various QC logs and plots (see the Materials and Methods). Features shown in Figure 1B are common in comparison with other existing QC tools whereas those in Figure 1C–E are mostly novel with respect to the *bamqc* module. We observe the per cycle substitution rate, which reflects cycle-specific mismatches (ideally equal for all cycles) (Figure 1C, upper panel) showing an increasing trend as the sequencing cycle progresses. In addition, there is a sudden rise in the number of substitutions observed in cycle number 62 where the substitution rate is 2.4% (average substitution rate is 1.87%), indicating a cycle-specific issue. Interestingly, for the same cycle in the bases where the mismatch is observed, the average quality drops sharply (Figure 1C, middle panel). We also observed that the average quality of mismatch bases decreases with the cycle, reflecting a significant inversely proportional relationship with substitution rate (*R* = –0.95; *P*-value < 2.2e-16). The occurrence of different substitutions, which should ideally be uniform for all cycles, shows cycle-specific variation (Figure 1C, lower panel); C > G/G > C substitutions have the lowest occurrence in comparison with the other substitution categories. A few other sample-specific features were also observed, such as: (i) C > A/G > T (10.3%) and A > C/T > G (10.3%) are the most prevalent changes (Figure 1D, upper panel); (ii) most of the observed substitutions are at low quality bases (Figure 1D, middle panel); (iii) the context defined by the duplet of bases, which precedes and succeeds the substitution, are also non-random. We observe that C_C and G_G are prominent contexts for A > C and T > G substitutions, respectively (Figure 1D, lower panel); (iv) overall, >15% of reads contain one mismatch (Figure 1E); (v) deletion events are more frequent than insertion events and A, T are the prevalent bases in single base InDels (Figure 1B); and (vi) clipping profiles for 5′ and 3′ read ends are similar (see Supplementary Results andSupplementary Figure S3 for more details).

The *genedepth* module estimates exon-wise DCOV to generate graphical plots and provides various depth-centric summary statistics. This module requires three files as input: an alignment file, a reference genome file and an exon-wise bed file. As a case scenario, to estimate *HRAS* gene coverage for the HG01402 data, we applied the *genedepth* module of the Mapinsights toolkit. A variability in depth is observed both within and across various *HRAS* exons (HG01402 was sequenced at 5× coverage, but *HRAS* exon 1 and exon 2 have low coverage in specific regions) (Figure 1F). This feature will enable researchers to probe deep into their exon/gene of interest, specifically in the context of different diseases, including cancer. See Supplementary Table S4 for more details.

Given genomic coordinates, the *siteinfo* module queries the position(s) in the alignment file and provides an extended set of quantitative information about the bases and reads mapped at that locus. Here we use *siteinfo* to query the coordinate ‘chr13:32906729–32906729’ on the HG01402 alignment file. Five reads are aligned in the query position with mapping quality 60, out of which four are second-in-pair and reverse strand reads. All the reads contain a ‘C’ base at that locus (where the reference base is ‘A’) with average base quality 25 (see Supplementary Table S5).

Lastly, *jumpreads* extracts all reads that show any one of the three following characteristics: (i) extra long inserts; (ii) read pair mapped to different chromosomes; or (iii) atypical alignment orientation (e.g. forward–forward, reverse–reverse, outward direction). This module takes an alignment file as the primary input to find reads with unusual mapping characteristics. We found ∼1.6 million (1.2% of total) reads with an insert size ≥ 1000 bp in the HG01402 alignment file using the module (see Supplementary Table S6 for more details).

### Mapinsights detects data quality issues across sequencing platforms

To compare and contrast sequence data quality across various platforms, Illumina [Hiseq, NovaSeq on which >90% of the world's genomic data have been generated (https://sapac.illumina.com/content/dam/illumina-marketing/documents/products/illumina_sequencing_introduction.pdf)], MGI-BGISEQ-500 (further referred to as MGI) and Element-Biosciences-AVITI (referred as AVITI), we have selected the most widely used community-recommended NA12878 WGS data that were sequenced using all the aforementioned platforms (some in different sequencing centers) for comparative analysis. HiSeq-2000/HiSeq-2500 (referred as HiSeq) and NovaSeq-6000 (referred as NovaSeq) fastq data were obtained from four different sequencing centers: the 1000G-platinum pedigree (PLPED) (sequencer: HiSeq-2000), the GIAB Consortium (sequencer: HiSeq-2500), 1000G-New York Genome Center (NYGC) (sequencer: NovaSeq-6000) and National Institute of Biomedical Genomics (NIBMG) (sequenced in-house using NovaSeq-6000 for this study). After initial pre-processing and alignment (see the Materials and Methods for details), the *bamqc* module of the Mapinsights tool was applied on all the NA12878 bam files.

**Figure 2.**
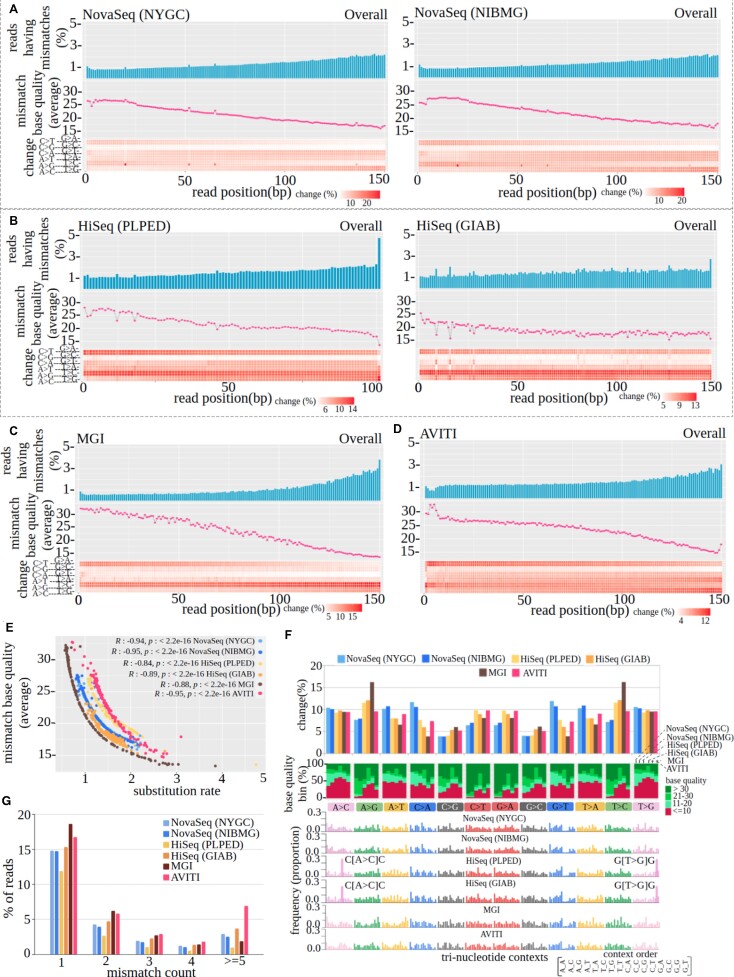
(**A–D**) *Per-cycle-profile* of NA12878 WGS data, generated using NovaSeq (in NYGC, NIBMG), HiSeq (in PLPED, GIAB), MGI and AVITI, respectively. (**E**) Scatter plot of the per cycle substitution rate versus mismatch base quality (average) of all platforms. (**F**) *Profile-of-substitution-categories* of NA12878 WGS data, generated using NovaSeq (in NYGC and NIBMG), HiSeq (in PLPED, GIAB), MGI and AVITI. (**G**) *Read-mismatch-content* of NA12878 WGS data, generated across all the platforms (as mentioned above).

The average substitution rate per cycle is higher in HiSeq (PLPED: 1.58%, GIAB: 1.49%) and AVITI (1.53%) compared with MGI (1.14%) and NovaSeq (NYGC: 1.37%, NIBMG: 1.30%) (Figure 2A–D). Pairwise comparison shows that the substitution rates are significantly different across orthogonal platforms (Supplementary Figure S4A). Although the average substitution rate is lowest in MGI, the end of reads of MGI sequences (last 25 bases) shows an inflated substitution rate in comparison with all other datasets (see Supplementary Figure S4A and Supplementary Table S7 for more details). For all sequencing data, the last base shows the highest substitution rate, reflecting cycle bias, except NovaSeq. As expected, an inversely proportional relationship (*R* ranges from –0.84 to –0.95 with *P*-value < 2.2e-16 across all platforms) is observed between substitution rate and mismatch base quality (average) where the former increases and the latter decreases along the read length (Figure 2E). We found bias in four fixed cycles in NovaSeq data (explained in detail in subsequent sections). We observed characteristic substitution patterns in different sequencing platforms for the same dataset; for example, in MGI, AVITI and NovaSeq data, C > T and G > A substitutions occur more in initial bases of the reads and decrease at the end. Further, for NovaSeq and AVITI data, A > G and T > C occur more in initial bases of reads, whereas for MGI data they occur more in the last few bases (Figure 2A, C, D, lower panel) reflecting a contrasting error pattern between sequencing platforms. Although not very prominent, few other trends are also present in the data. We noticed a preponderance of certain mismatches in cycles where the substitution rate is higher (cycle bias) in HiSeq (GIAB) data (Figure 2B).

Further, a considerable variability is observed in substitution percentages in NA12878 whole-genome samples sequenced using different platforms (Figure 2F, upper panel) reflecting sequencing run-, center- or platform-specific variation that can easily be quantifiable from Mapinsights outputs. The major changes are C > A/G > T (10.3/11.8%) in NovaSeq-NYGC, A > T/T > A (10.6%/10.8%) in NovaSeq-NIBMG and C > T/G > A (9.7%/9.6%) in AVITI data, whereas A > G/T > C (11.4% in HiSeq-PLPED, 12% in HiSeq-GIAB and 16.1% in MGI) are the predominant mismatches in HiSeq and MGI data. A > G/T > C transitions are strikingly higher in MGI data, >4% higher compared with HiSeq data, even larger than any substitutions in any other sequencing platforms. We have seen a read-pair and strand-specific disparity in these changes in MGI data. A > G (24.7%) substitutions occur almost 14.7% more in the second-in-pair and in the forward strand than T > C (10%), and the pattern is opposite in the reverse strand where T > C (24.8%) occurs more and the deviation is 14.8%. However such a deviation is lower in first-in-pair reads [forward: A > G (17.9%) T > C (11.4%) and reverse: A > G (11.4%) T > C (17.9%)] (Supplementary Figure S4B). For the same platform (MGI), we also observed that the same substitutions (A > G/T > C) occur more in certain contexts [GAG > GGG (14%) and TTT > TCT (14%)] which is not prominently seen in other platforms [G_G (∼6%), T_T (∼9%) in AVITI, G_G (∼11%), T_T (∼6%) in NovaSeq and HiSeq]. These context, strand and paired-end issues related to elevated A > G/T > C changes indicate the existence of sequencing artifacts. However, these elevated transitions do not have any major impact on variant calling (Supplementary Figure S4C); the artifacts are probably randomly distributed.

We found that a large proportion of C > T/G > A and A > G/T > C mismatches have high base quality in NovaSeq data compared with other platforms (Figure 2F, middle panel). A bias is observed in A > C and T > G changes where >50% of such changes have low base quality in all the platforms except NovaSeq. An enrichment of a particular nucleotide context is also noticed in A > C and T > G mismatches where C_C (i.e. CAC > CCC) and G_G (i.e. GTG > GGG) occur significantly more often (*P*-value < 0.002, Dixon's test for outlier) compared with the other nucleotide context (Figure 2F, lower panel) in HiSeq data, reflecting context-dependent bias.

Further, *read-mismatch-content* shows a noticeable variability across sequencing platforms. Reads having a single mismatch are higher in MGI (18.6%) and AVITI (16.7%) compared with HiSeq (PLPED: 11.8%, GIAB: 15.3%) and NovaSeq data (NYGC: 14.8%, NIBMG: 14.7%). In AVITI data, we observed a large number of reads (6.9%) containing ≥5 mismatches which is 2-fold higher compared with other platforms (Figure 2G), reflecting sequencing issues.

### Detection of a unique error pattern pertaining to NovaSeq whole-genome data

**Figure 3.**
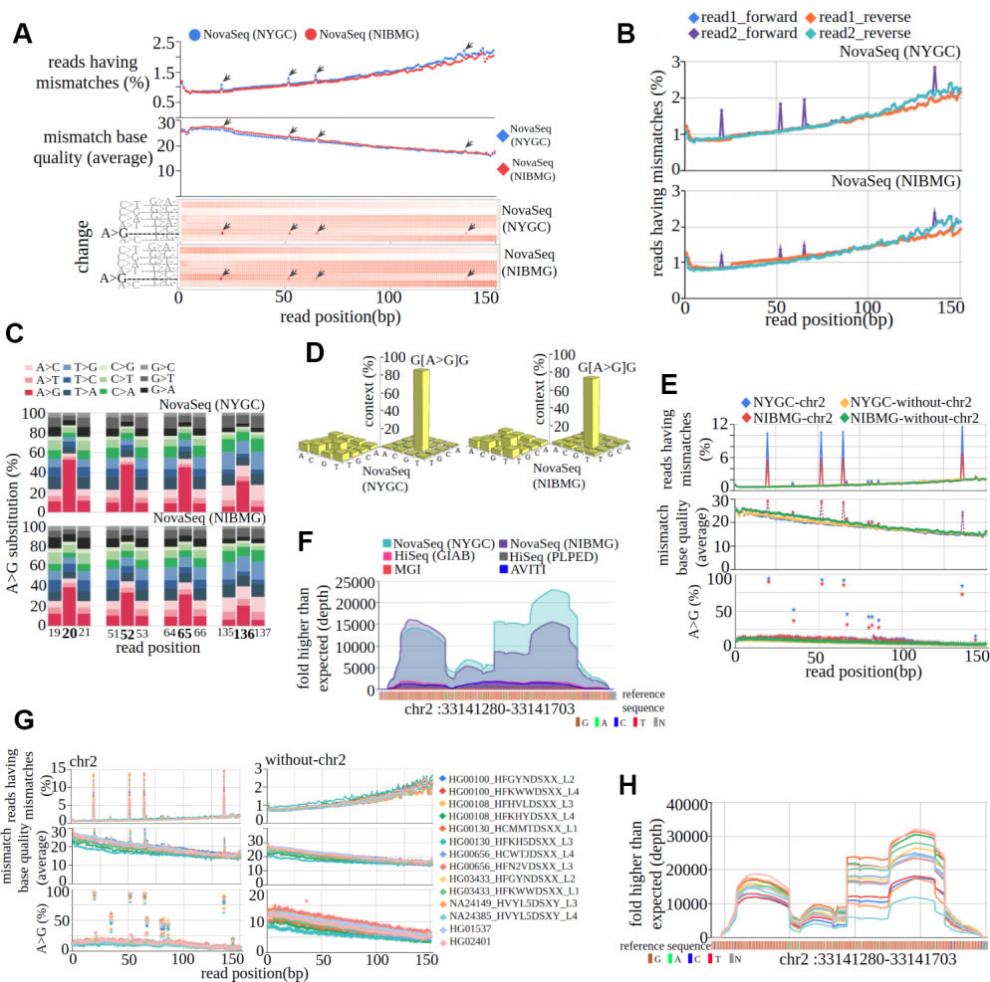
(**A**) *Per-cycle-profile* of NA12878 WGS data, generated using NovaSeq in NYGC and NIBMG. (**B**) *Per-cycle-profile* of NA12878 WGS data, generated using NovaSeq in NYGC and NIBMG in paired-end orientation. (**C**) Percenage of different substitution categories in and around cycle numbers 20, 52, 65 and 136 present in read2 forward strand reads. (**D**) Nucleotide context of A > G change present in read2 forward strand reads in the NA12878 sample sequenced in NYGC and NIBMG. The plot next to the right side of the *y*-axis shows the distribution of 16 different contexts of A > G found in four affected cycles, and that on the left side of the *y*-axis shows the distribution of 16 different contexts of A > G found in non-affected cycles. (**E**) A comparative view (only chromosome 2 sequences versus all sequences except chromosome 2 sequences) of the *per-cycle-profile* of NA12878 whole-genome data, generated using NovaSeq in NYGC and NIBMG. (**F**) Sequencing center-wise normalized DCOV of a genomic segment of chromosome 2 (hg19, chr2: 33141280–33141703). To validate the cycle-specific error pattern present in chromosome 2 observed in the NA12878 sample sequenced in two different labs (NYGC and NIBMG), we have used additional WGS datasets generated from NYGC and NIBMG using NovaSeq sequencer. The Mapinsights *bamqc* module was run on these additional datasets. (**G**) *Per-cycle-profile* of additional whole-genome datasets. Individual samples were marked in different colored lines. (**H**) Normalized DCOV status of a genomic segment of chromosome 2 (hg19, chr2: 33141280–33141703) in additional datasets.

Mapinsights was able to identify an extremely non-random systematic bias, sudden spikes or an increased proportion in substitution rate, in four fixed cycles (cycle numbers 20, 52, 65 and 136) in NovaSeq data independent of sequence data generation sites (NYGC and NIBMG) (Figure 3A). This cycle-specific spike in substitution rate was absent in Hiseq, MGI and AVITI data (Figure 2B–D). In general, the substitution rate is inversely proportional to mismatch base quality (Figure 2E, see also Figure 1C for HiSeq data) which can be seen in all cycles except the above-mentioned four cycles where the pattern is opposite (Figure 3A, middle panel). An over enrichment of A > G substitution is observed in these four cycles (Figure 3A, lower panel). Further analysis found that A > G substitutions occur only in the forward strand and the second read (read2) in paired reads sequencing (Figure 3B). The percentage of A > G substitution occurring in these four cycles in the read2 forward strand reads ranges from 31.2% to 52.8% in NovaSeq-NYGC data and from 20.0% to 38.7% in NovaSeq-NIBMG data, which is significantly (*P-*value < 0.002, Dixon's test for outlier) higher compared with the adjacent cycles (Figure 3C). Both percentage of A > G substitution and their quality are higher in the initial cycle (cycle number 20) and decrease subsequently in cycle nos 52, 65 and 136. Further, we have compared the occurrence of 16 possible nucleotide contexts of A > G substitutions that were specific to the four affected cycles, and G_G context is found to be significantly higher (*P*-value < 0.002, Dixon's test for outlier) in comparison with other contexts. To find the significance of this observation, we calculated the context distribution of A > G substitution present in cycles other than the four affected cycles, and find that the G_G (NovaSeq-NYGC: 86%, NovaSeq-NIBMG: 74%) motif is significantly higher (*P*-value < 0.002, Dixon's test for outlier) in affected cycles (Figure 3D). So, it is likely that GAG motifs are erroneously converted to GGG during sequencing. To understand how randomly these four cycle-specific errors are distributed across different parts of the genome, our deep analysis found the errors are present only in chromosome 2 (Figure 3E). An extremely G-rich segment of chromosome 2 (hg19, chr2: 33141280–33141703) was found to be an erroneous region that mostly contributed to the cycle-specific error pattern in NovaSeq data. A substantial amount of NovaSeq reads [>0.14% (NYGC) and > 0.10% (NIBMG) of total mapped reads which is a >5000 to 10,000-fold higher than expected DCOV in the studied samples] were aligned to this region which is significantly (*P*-value < 0.00001, *Z*-test of proportions) higher compared with HiSeq, MGI and AVITI data (Figure 3F).

Since the error pattern is not observed in HiSeq, MGI and AVITI data, it is not a sample-specific or data processing problem. As the observation is consistent in NovaSeq data generated in two different labs, the possibility of particular sequencing run-/lab-specific problems can be ruled out. We suspect that the problem pertains to the NovaSeq sequencing process. In order to test our assumption, we have performed an analytical experiment. We applied the *bamqc* module on three categories of data sequenced using the NovaSeq sequencer comprising nine different whole-genome samples. Category 1: data of five whole-genome samples sequenced in different sequencing machines, different flow cells and lanes that were downloaded from NYGC, and data were process using the NIBMG pipeline; category 2: whole-genome alignment data (aligned against GRCh38) of two samples sequenced and processed using the NYGC pipeline; and category 3: data of two whole-genome samples sequenced and processed using the NIBMG pipeline. The same error pattern was found in all these additional data (Figure 3G, H; Supplementary Figure S5), suggesting that our initial assumption is correct. However, since the region is very small, it does not have much effect on the variant calling. Further, we note that this pattern of error was detected in NovaSeq whole-genome data with a read length of 150 bp. Publicly accessible WGS data on the NovaSeq platform for other read lengths are not available for assessment of the same error pattern.

### Detection of outlier samples based on sequencing artifacts from 1000G-exome samples

**Figure 4.**
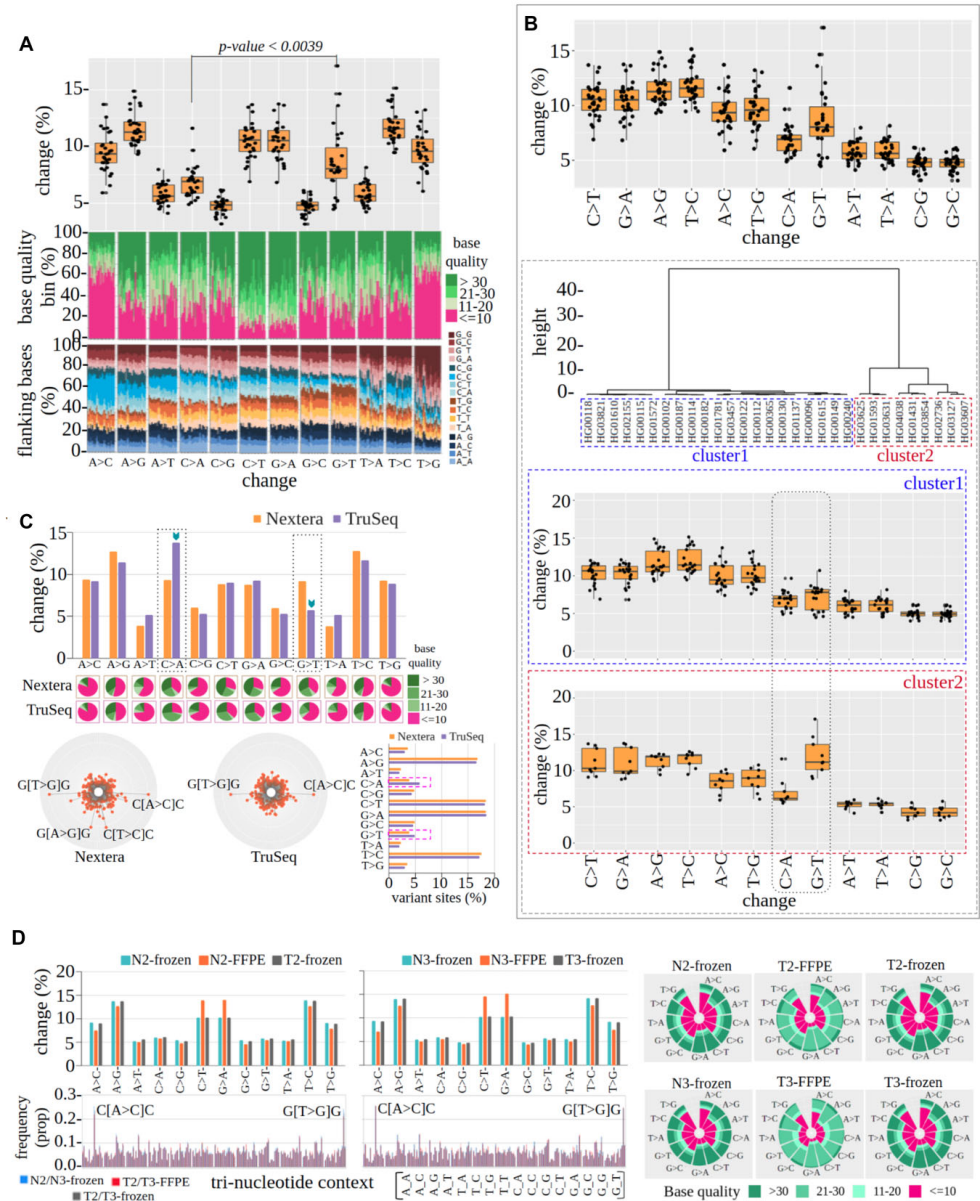
(**A**) Combined representation of substitution profile for 30 exome samples from the 1000G-phase3 project [each dot (upper panel) and vertical line (lower panels) represents an individual sample]. (**B**) Multisample cluster analysis based on subfeature (distribution of substitution categories) of *profile-of-substitution-categories* from the *bamqc* results of 30 exome samples reflects the outlier group (upper panel) and batch plot of substitution categories of both the clusters (lower panels). (**C**) Substitution profile of Nextera and TruSeq data (upper panel), quality distribution (middle panel) and nucleotide context (lower panel). Horizontal bar charts represent the result upon running GATK-HaplotypeCaller on both types of data, highlighting the error profile observed for C > A variant calls. (**D**) Substitution type, quality distribution and nucleotide context profiles with respect to three types of data [formalin-fixed paraffin-embedded tumor tissue (T2-FFPE, T3-FFPE), fresh frozen normal (N2-frozen, N3-frozen) and tumor tissue data (T2-frozen, T3-frozen)] of two lung adenocarcinoma patients.

To investigate whether Mapinsights features can be used to identify outlier samples we applied *bamqc, multisample-bamqc* and *batchplot-bamqc* modules of Mapinsights on open-source sequencing data. In a dataset of 30 exome samples (source: 1000G-phase3), a significant (*P*-value < 0.0039, Mann–Whitney U-test) deviation is observed in G > T and C > A substitution pairs (which ideally should be similar) with a median of 8.0 and 6.9 (average 8.6 and 6.8), respectively, reflecting a strand-specific error incorporation probably during library preparation known as oxo-G artifacts (Figure 4A, upper panel). Further, to detect the outlier samples, we applied *multisample-bamqc* and *batchplot-bamqc* on the same dataset. In general, transition pairs (e.g. C > T:G > A) and transversion pairs (e.g. A > T:T > A) are expected to show similar trends in proportional change. Large deviation between these pairs reflects biases in sequencing data. Therefore, we used the difference in proportional change between substitution pairs as a metric to detect outliers. Based on the difference in pairs, we perform hierarchical cluster analysis. The Euclidean method is used to calculate the distance followed by clustering using Ward's method with default parameters.

We found two major clusters, one comprising 21 samples (cluster1) with the other nine samples grouped in another cluster (cluster2) (Figure 4B). From comparative analysis using *batchplot-bamqc* output, it is evident that samples belonging to cluster2 show a notable erroneous property where the deviation is significant (Figure 4B, lower panel) compared with cluster1. These nine samples are detected to be outliers. Further, we ran GATK-HaplotypeCaller (59) on all 30 samples, to check whether this technical bias has any effect on variant calls. A significant difference between G > T (median raw: 3.56%; filtered: 3.48% across 30 samples) and C > A (median raw: 3.44%; filtered: 3.38% across 30 samples) is found to be present in the raw variant call set (Wilcoxon *P*-value = 0.01) and filtered (Wilcoxon *P*-value = 0.005) variant call set in nine samples (cluster2) (Supplementary Figure S6). In the filtered data, a deviation of 0.09% (median) between C > A and G > T is noticed in nine outlier samples. We also observed deviation in another 21 samples (cluster1); however, this was not significant (Supplementary Figure S6).

We also applied the *bamqc* module on NA12878 exomes, sequenced using two different library capture kits (TruSeq and Nextera) and FFPE samples. A prominent difference is observed in the substitution pair (C > A and G > T) in NA12878 exomes. C > A (13.7%) transitions are predominant in TruSeq data, and are >2-fold higher compared with G > T (5.7%) substitutions, indicating the same error source, as mentioned above (Figure 4C). Further, to determine whether the preponderance of C > A errors has any impact on variant calling, we ran GATK-HaplotypeCaller on NA12878 exome sequencing data. After removing artifacts from variant call sets by applying recommended hard filters, it emerged that indeed in filtered variant calls, C > A (5.8%) substitutions are more frequent than G > T (4.9%) substitutions in TruSeq and also higher in number compared with Nextera data (C > A and G > T both 3.8%) (Figure 4C, lower panel bar plot). Also, ∼0.9% deviation is observed between C > A and G > T in the filtered variant calls. C > T and G > A substitutions are found to be major changes (>13.9%) in both FFPE data, reflecting the known error signal associated with FFPE (Figure 4D).

Furthermore, we noticed an abundance of low-quality (≤10) A > C and T > G mismatches in 30 exome samples, NA12878, FFPE and other exome samples, mostly in the nucleotide context C_C and G_G, respectively (Figure 4A, C, D). Overall the base quality of FFPE data is lower compared with its paired samples (Figure 4D, right panel). We also found that read2 contains more mismatches in comparison with read1 in sequence data across all the platforms (Supplementary Figure S7), indicating general sequencing issues associated with sequencing chemistry.

### Mapinsights identifies distinctive features of reads that are associated with low confidence variant sites

Identification of false-positive variant calls is of utmost importance to improve the downstream interpretation of genome sequencing datasets. Specific features of the Mapinsights tool can identify outlier samples, quality- and sequence artifact-related issues from sequencing reads. To demonstrate the importance of such features in detection of artifact variant sites, we used community standard open source variant call sets generated using state-of-the-art analytical pipelines. Variant call files of NA12878 were downloaded from GIAB (benchmarked variant list v.3.3.2) and NYGC for the analysis. We also downloaded the corresponding sequence alignment file (cram file) of NA12878 from NYGC (see Supplementary Table S8 for details). We included only passed single nucleotide variant (SNV) sites from the NYGC variant call set and compared that with the GIAB benchmarked SNV callset. In order to estimate chromosome-level variation, six randomly selected chromosomes were analyzed separately.

**Figure 5.**
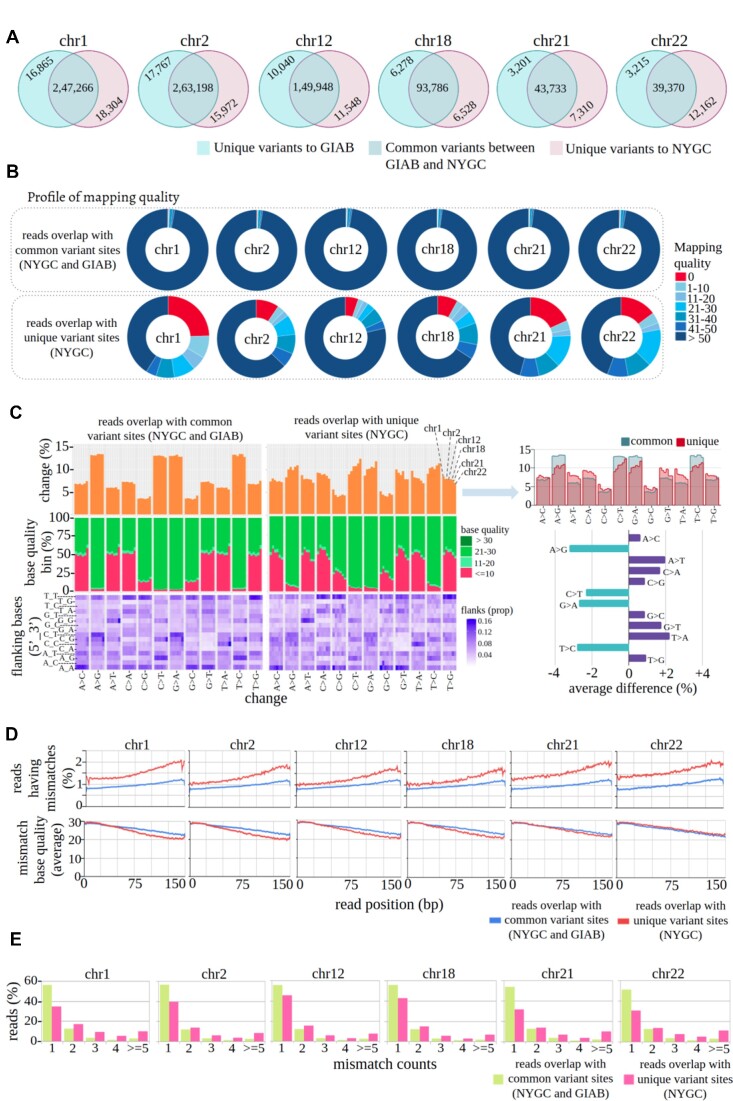
(**A**) Venn diagram representing the similarity and dissimilarity among SNVs between NYGC- and GIAB-benchmarked variant calls for the NA12878 sample in six different chromosomes. The majority of the variant sites are common between NYGC and GIAB calls. (**B**) Profile of mapping quality between reads that overlap with *common-variant-sites* (variant sites common between NYGC and GIAB) and *unique-variant-sites* (unique to NYGC). (**C**) *Profile-of-substitution-categories* between reads that overlap with *common-variant-sites* and *unique-variant-sites* [six randomly selected chromosomes were analyzed to estimate variation (if any) among them; each vertical line represents an individual chromosome]. (**D**) Profile of per cycle mismatch rate between reads that overlap with *common-variant-sites* and *unique-variant-sites*. A clear difference is observed where the per cycle mismatch rate is always higher in reads that overlap with *unique-variant-sites* compared with *common-variant-sites* for each cycle and each chromosome. **(E**) Profile of *read-mismatch-content* between reads that overlap with *common-variant-sites* and *unique-variant-sites*. Reads having ≥2 mismatches are always higher in *unique-variant-sites*, whereas one mismatch reads are higher in *common-variant-sites*.

The majority of the NYGC variants (∼93%, median of six chromosomes) overlap (further termed as *common-variant-sites*) with GIAB-benchmarked calls and ∼7% (median; six chromosomes) of variants are found to be unique to NYGC (further termed as *unique-variant-sites*). If GIAB is the ‘gold standard’, then the *unique-variant-sites* are likely to be errors. The *unique-variant-sites* had a relatively wide range (∼5% to ∼23%) spanning across the six different chromosomes, with chromosome 22 having the highest proportion (∼23%) (Figure 5A). All reads mapped to *unique-variant-sites* and *common-variant-sites* were extracted from the NA12878 alignment file and we applied the *mapinsights-bamqc* module. We found distinct and undetected features, predictive of a low-quality artifactual variant, in different modules of Mapinsights, including *mapping-quality*, *profile-of-substitutions-categories*, *per-cycle-profile* and *read-mismatch-content*.

For all the chromosomes, a large proportion (∼18%, median) of reads having low mapping quality (∼12% reads with MapQ score 0, ∼4% with 1–10, ∼2% with 11–20) are observed in reads mapped to *unique-variant-sites*. whereas most (∼97%, median) of the reads have mapping quality >50 in *common-variant-sites* (Figure 5B). Compared with *common-variant-sites*, the A > T/T > A and C > A/G > T transversions are observed more (∼2%) in reads that overlap with *unique-variant-sites* (Figure 5C, top panel). Similarly, an increased proportion of low quality (≤10) C > G/G > C mismatches is found to be present in *unique-variant-sites* (Figure 5C, middle panel). The trinucleotide context also shows differences for *common-variant-sites* and *unique-variant-sites* (Figure 5C, bottom panel). We identified considerable differences in the *per-cycle-profile* (Figure 5D). For each cycle, a sharp difference is observed in the substitution rate of reads that overlap with *common-variant-sites* and *unique-variant-sites*. The substitution rate is always higher in reads that overlap with *unique-variant-sites* in all six chromosomes; however, such deviation is not found in per cycle mismatch base quality (Figure 5D). We also observed a prominent difference in *read-mismatch-content* profiles. Reads containing ≥2 mismatches are always higher in reads that overlap with *unique-variant-sites* compared with *common-variant-sites* (Figure 5E).

Next, we performed a logistic regression-based analysis to test the usability of the Mapinsights features in discriminating ‘high-confidence’ benchmark variant sites (*common-variant-sites*, see above) from ‘low confidence’ variant sites. We consider various attributes: *read-mismatch-content*, read clipping status, read InDel counts, reference and variant base homopolymer counts, read mapping quality and insert size as independent predictor variables for this analysis. A total of ∼128 000 sites comprising benchmarked (∼64 000) and non-benchmarked (∼64 000) sites were included, out of which 70% (∼89 000) sites were used to find the best parameter estimates. The remaining 30% (∼38 000) are then used to evaluate the accuracy of the model. For heterozygous sites, the regression analysis can segregate benchmark from non-benchmark sites with an accuracy of ∼81.5%, whereas for other allele homozygous sites the accuracy is ∼72.68% (see Supplementary Table S9 for more details), reflecting a potential applicability of Mapinsights features to filter out possible erroneous variant calls.

### *Per-cycle-profile* reveals cycle-specific substitution bias and new error patterns

**Figure 6.**
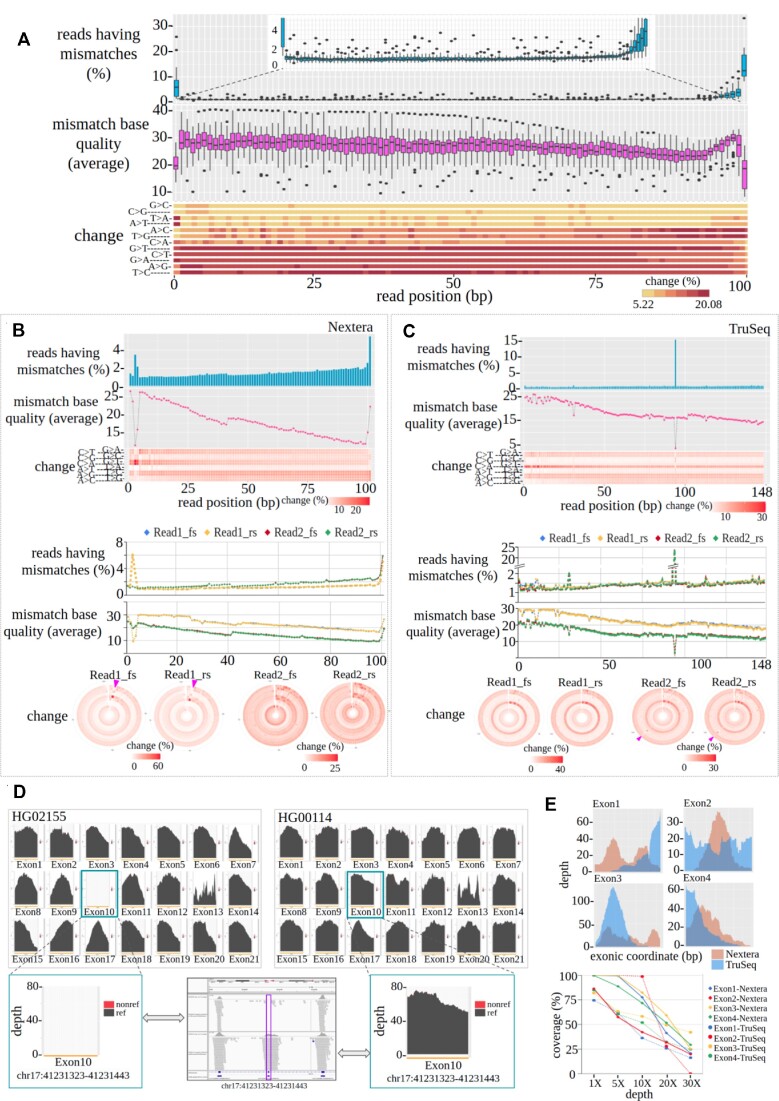
(**A**) Combined representation of the *per-cycle-profile* for 30 exome samples from the 1000G-phase3 project. Both per cycle mismatch rate and mismatch base quality are represented with box plots. The lower panel heatmap represents the occurrence of different substitution categories. (**B** and **C**) *Per-cycle-profile* of Nextera and TruSeq data in an overall (top plot) and paired-end orientation (lower plot) manner. (**D**) The upper block shows exon-wise coverage plots for the *BRCA1* gene in HG02155 and HG00114 samples of the 1000G-phase3 project generated using the Illumina Hiseq platform. The *y*-axis of each plot represents the depth, and the *x*-axis represents the exonic position. The lower block shows an enlarged view of exon 10 where a significant variability is observed between samples in DCOV, and a validating IGV snapshot was provided. (**E**) Exon-wise DCOV plot for the *CDKN2A* gene generated using two different types of capture kit - Nextera and TruSeq (upper four plots) and exon-wise fraction covered at different depths (lower plot).

We performed aggregate analysis and found that the per cycle average substitution rate is ∼1.40 for whole-genome samples (range 0.7–4.7). For the exome, it is ∼1.27 (estimated from 30 exome samples; range 0.53–33.3). The end cycles are enriched with error (evident from an increase in substitution rate) and low quality, as observed from different datasets (Figure 6). A contrasting example is shown in Supplementary Figure S8A where sample HG00187 is severely affected by cycle bias compared with sample HG01431. Several cycle-specific patterns are found in the 30 exome data where the occurrence of C > T, G > A, A > G and T > C transitions decreases as the cycle progresses as opposed to A > C, T > G transversions where the pattern appears to be the opposite (Figure 6A). Further sample-wise estimates also reflect the same pattern (Supplementary Figure S8B). G > T changes are consistently distributed across cycles (except the last 4–5 cycles) and occur more frequently compared with C > A. Further, A > T/T > A substitutions are higher in the first base which is unusual and likely to be artifacts. Our sample-level analysis is able to detect a high rate of G > T substitutions in specific (6 out of 30) samples (Supplementary Figure S8B).

We further evaluated the effect of different sequence capture kits (Illumina-Nextera and Illumina-TruSeq) to find characteristics differences in terms of *per-cycle-profile*. We investigated NA12878 exomes which were sequenced using two different capture kits. Both sets of data were sequenced in two different lanes. Cycle numbers 3 and 4 (contain mostly C > A/G > T mismatches) and the last cycle have a significantly high (*P-*value < 0.002, Dixon's test for outlier) substitution rate compared with the nearby cycles in Nextera data (Figure 6B), whereas a single cycle (cycle number 95) is severely affected with a significantly higher error rate (*P-*value < 0.002, Dixon's test for outlier) in TruSeq, reflecting that heavy substitution cycle bias exists in the data (Figure 6C). A sharp drop is observed in affected cycles in both capture datasets. We found that the bias occurs only in the first-in-pair read (read1) in the case of Nextera (Figure 6B) whereas the second-in-pair read (read2) was affected in TruSeq data (Figure 6C). Our detailed analysis found that in Nextera data both lanes had the same issues (Supplementary Figure S8C), indicating a global issue, whereas in TruSeq data such issues appeared only in one lane (lane 2) (Supplementary Figure S8C), reflecting a local lane-specific issue. For both cases (Nextera and TruSeq), the issues are associated with sequencing run. In FFPE data, C > T/G > A occurs more in the initial cycles and decreases subsequently in the following cycles (Supplementary Figure S8D). In a nutshell, Mapinsights is able to identify different types of sequencing artifacts in various data types.

### Exon-wise gene depth profiles reveal unevenness in sequencing coverage

To evaluate whether the *genedepth* module can capture depth-related issues for a gene/exon, we considered targeted sequencing data. The *genedepth* module was applied on two sets of exome sequencing data to explore the exon-wise depth profile: (i) 1000G-phase3 exomes (*n* = 30) and (ii) NA12878 exomes (*n* = 2), sequenced using two library preparation kits (Nextera and TruSeq). We estimated exon-wise DCOV for the *BRCA1* gene (all 21 exons) in all 30 exomes. To demonstrate the usefulness of the module, we explained the results with respect to two samples, i.e HG00114 and HG02155. As shown in Figure 6D, the DCOV varies across exons and is not even uniform over the entire region of an exon itself. For certain exons, the variability is noticeable, such as exon 13 in both the samples and in other samples as well (Supplementary Figure S9A). Strikingly, we see that the mean DCOV is 0 for exon 10 in the HG02155 sample whereas it is 70× in the other. Since the contrast seems unusual, we validated the observation using IGV and found that our results are indeed correct and no reads mapped to that particular exon (Figure 6D, IGV snapshot: marked in a violet box). Most samples (24 out of 30 samples) have bad coverage for exon 10 of the *BRCA1* gene (Supplementary Figure S9B). This type of event (whole-exon deletion) or issue (capture efficiency of design probes) can easily be detected from the output of the *genedepth* module.

Next, we studied the *CDKN2A* gene using the second dataset which was sequenced using two different library kits. Both library kits suffer from non-uniformity across different exons of *CDKN2A* and substantially large regions of specific exons have very little or no depth at all (Figure 6E). Detailed statistics are provided in Supplementary Table S10. Comparative analysis on NA12878 exomes sequenced using the two library kits shows that ∼55% (median) regions are covered at 10× depth across all exons in TruSeq whereas in Nextera coverage is ∼75%, indicating better consistency in sequence coverage. However, at 30× depth, almost all the exons of *CDKN2A* perform equally badly in both datasets by covering <30% of the targeted regions (Figure 6E).

## DISCUSSION

Evaluating the quality of HTS data is complex and challenging. A systematic and in-depth QC analysis at various data processing steps is essential. Most of the existing QC tools lack methodical attention to data quality in terms of sequenced base mismatches with respect to reference nucleotides. To fill this knowledge gap, we developed the Mapinsights toolkit that facilitates multiple functionality regarding quantitative assessment of data quality at a deeper level. Multiple modules are developed as part of Mapinsights such as: *bamqc* which performs QC analysis of an alignment file that includes common and novel QC features*; multisample-bamqc* and *batchplot-bamqc*, enabling comparison analysis across multiple samples that allow evaluation of consistency among samples and outlier detection; *genedepth* which estimates exon-wise DCOV for genes*; siteinfo* which queries the genomic position in the alignment file to obtain site-specific information; and, lastly *jumpreads* which extracts all the reads having atypical alignment properties. Mapinsights is open source and can easily be installed and integrated in the HTS analytical pipeline and run comparably/faster than the existing QC tools with a low memory footprint. Mapinsights provides QC metrics in a modular way in either text, pdf or HTML format.

Analysing various kinds of open-source data (exome and whole genome) using Mapinsights, we identify outlier samples in 1000G-phase3 datasets with respect to sequencing artifacts where G > T substitutions are significantly higher then C > A substitutuions (ideally they should be equal), suggesting an inflated rate of artifacts probably being oxo-G errors which are known to occur due to heat, shearing and metal contaminants, where the G base is oxidized and mispairs with adenine leading to G:C to T:A mismatches (21,22).

As expected, there is an inverse proportional relationship between per cycle substitution rate and mismatch base quality (average), where the former increases as the sequencing cycle progresses. This indicates that the end of the reads contains more mismatches which are primarily known to be related to phasing and pre-phasing problems in Illumina platforms (29). In every sequencing cycle, inadequate flushing of non-incorporated nucleotides and improper removal of the blocker result in phasing and pre-phasing events that influence the overall light signal of a cluster. Initially the effect is low, but as the cycle progresses the amalgamation of these events causes pale cluster signals, leading to a lower base quality and higher error rate towards the end of the read (29,32). In addition, adapter content and erroneous end repair also contribute to elevated error rates at the end of the reads. We detected substitutional cycle bias where one or more cycles have an elevated error rate compared with adjacent cycles with a drop in base quality. In general, a drop in base quality is also observed in relation to the affected cycles which are assumed to be due to the problems of fluidics during the Illumina sequencing run (60). Clogs and leaks in the fluidics line lead to bubbles and long air gaps, which in turn lead to poor data from clusters of one or many tiles and cycles. Similarly, the sequencing cycle can also be affected by freely moving chemistry crystals, dust or lint particles. In such cases, the signal of one or several cycles becomes polluted, resulting in poor imaging (60,61). This explains the cycle-specific sudden drop in quality and higher error rate which are observed from the QC report of open-source data generated by the Mapinsights tool. We identified cycle-specific non-random mismatch patterns.

We also observed various errors and biases in the recently launched sequencing platforms (MGI and AVITI), such as end of the reads containing more mismatches, cycle bias with drop in quality, non-random substitution patterns in cycles, context and quality issues. Some of the error patterns are similar to those in Illumina. However, some observations are specific to recent platforms. For instance, A > G and T > C mismatches in MGI data (which have context, strand and pair-end specificity) and reads having ≥5 mismatches in AVITI data are higher compared with other platforms, reflecting additional errors and biases. However, the underlying mechanism of the errors and biases observed in these platforms are not yet known and will require further research, experiments and in depth exploration of sequencing methods and techniques.

Further, Mapinsights is able to detect issues involved in the NovaSeq data for 2 × 150 bp reads. A high G-rich region of chromosome 2 (chr2: 33141280–33141703) turns out to be a universal erroneous region for NovaSeq data, where a substantial number of reads mapped to this small region compared with other platforms. One plausible explanation of why this is observed in NovaSeq could be due to G overcalling. NovaSeq uses two-channel SBS chemistry where T, C and A bases are labelled using red, green and both colors. However, the G base is permanently dark and that creates a problem. For instance, the machine cannot distinguish between no signal caused by sequencing issues versus absence of signal for G bases, resulting in overcalling of G base with higher base quality, and also poly(G) causes substitution error (35) (https://sequencing.qcfail.com/articles/illumina-2-colour-chemistry-can-overcall-high-confidence-g-bases/). As previously reported, our tool is also able to find that there is an abundance of A > C mismatches in HiSeq data (17,29,31), and read2 sequences are prone to more errors compared with read1 in Illumina sequence data (32,34). We also observed known artifacts, such as elevated C > T/G > A mismatches, in FFPE data compared with fresh frozen tissue (56). Our analysis shows that Mapinsights can identify outlier samples, quality- and sequence artifact-related issues that are specific to exome sequencing, tissue preservation and library preparation which impact on variant calling.

We show that Mapinsights can identify distinct properties of reads that overlap with non-benchmarked variant sites, or sites which are likely to be errors. A substantial difference is observed in *mapping-quality*, *profile-of-substitution-categories*, *per-cycle-profile* and *read-mismatch-content* when compared between reads that overlap with benchmarked and non-benchmarked variant sites. Logistic regression analysis showed that Mapinsights features are capable of separating both the classes (benchmark and non-benchmark) with high accuracy. However, the accuracy could be further improved upon applying a deep learning approach such as the sophisticated state-of-the-art tools DeepTrio (62) [version of DeepVariant (63) with extended functionality] and DeNovoCNN (64) that employs a convolutional neural network (CNN) to classify variants (true variant versus sequencing artifact). These tools are trained with known sequencing errors, technical artifacts, mapping errors, etc. For instance, DeNovoCNN includes manually verified noisy regions (a lot of technical errors) during training to make the model more robust and accurate. Since new chemistry or technology often comes with its own error pattern, we anticipate that our tool could aid in understanding such new errors or biases on a deeper level and that will be helpful in such training. Our tool successfully portrays the existence of variability in depth of coverage across different exons of a gene, reflecting a chance that potential mutation could be missed due to insufficient depth of coverage, and assists in identification of the biological events (structural variation) or issues (capture efficiency of design probes).

Finally, along with novel features, Mapinsights brings together functionality of multiple existing QC tools that facilitate in-depth and novel understanding of sequence data quality and sequencing artifact-related issues. Mapinsights can rapidly assess the variability observed in QC profiles of genome-scale sequence data generated using various sequencing platforms. Our tool is efficient in comparing and identifying outlier samples based on certain new and existing features. Further, Mapinsights also identifies unique features of reads around low confidence variant sites that help in filtering out such sites with high accuracy. Additional functionalities enabled in Mapinsights, including querying genomic coordinates in the alignment file, extracting jump reads and exon-wise gene depth, make it more versatile and resourceful.

## Supplementary Material

gkad539_Supplemental_File

## Data Availability

Datasets generated in NIBMG for this study are available from the European Genome-Phenome Archive under accession code PRJEB48610 or the corresponding author upon request. Mapinsights source code is publicly available in GitHub at https://github.com/subrata-codeons/mapinsights (permanent DOI:10.5281/zenodo.8009589).
