# Supplementary material for: Mapinsights: deep exploration of quality issues and error profiles in high-throughput sequence data
Source: Nucleic Acids Res. 2023 Jun 28;51(14):e75. doi: 10.1093/nar/gkad539 (PMC10415152; doi:10.1093/nar/gkad539)
Supplement: gkad539_Supplemental_File [file gkad539_supplemental_file.pdf]

Mapinsights: deep exploration of quality issues and error profiles in high-throughput sequence data

Subrata Das, Nidhan K. Biswas*, Analabha Basu*

Supplementary Information

Contents

Supplementary Methods 2-3

Supplementary Results 4-5

Supplementary Figures

Figure S1. Flowchart of *bamqc* module of Mapinsights 6
Figure S2. Flowchart of *genedepth*, *siteinfo* and *jumpreads* module 7
Figure S3. Paired-end orientation results of HG01402 sample 8
Figure S4. Comparison of QC features and variant calls across sequencing platforms 9
Figure S5. Validation of cycle specific substitution bias in NovaSeq data 10
Figure S6. Deviation between C>A and G>T in variant calls of 30 exomes 11
Figure S7. Profiles of *read mismatch content* across different datasets 12
Figure S8. Per cycle profile of different sequencing datasets 13
Figure S9. Exon-wise coverage plot of *BRCA1* gene in two samples 14

Supplementary Tables

Table S1. Features comparison across multiple QC tools.. 15
Table S2. FASTQ, BAM, VCF file download sources 17
Table S3. Run time of Mapinsights modules... 18
Table S4. Exon-wise depth status of *HRAS* gene in HG01402 sample............................ 19
Table S5. Output of Mapinsights *siteinfo* module on a variant site of *BRCA2* gene.......... 20
Table S6. Metrics generated by Mapinsights *jumpreads* module................................ 21
Table S7. Descriptive statistics of substitution rate across sequencing platforms............ 22
Table S8. Download source of NA12878 CRAM and VCF 23
Table S9. List of predictor variables,logs and results of logistic regression analysis......... 24
Table S10. Exon-wise depth status of *CDKN2A* gene 25

Supplementary Methods

Library preparation and sequencing.

Whole genome libraries were prepared using the TruSeq Nano DNA Library Preparation Kit (Illumina) with 100ng of good quality genomic DNA (assayed by nanodrop-based spectrophotometric and Qubit based fluorometric method) in accordance with the manufacturer's instructions. Briefly, genomic DNA was sheared using the Covaris sonicator to a target size of 350 bp followed by end-repair, selection of library size, adenylation and adapter ligation steps. Further, DNA fragments were amplified using PCR. The amplified DNA libraries were checked in Agilent 2200 TapeStation (Agilent Technologies) using high sensitivity D1000 ScreenTape, quantified using Qubit based fluorometric method and Real Time PCR, normalised and subjected to equimolar pooling. Finally the pooled libraries were loaded on S4 flow-cell (illumina) and sequenced on an Illumina NovaSeq 6000 instrument using 2 x 150bp cycles protocol.

Speed and memory requirement

Since, the number of features and their computational complexities are variable across different QC tools therefore it is not appropriate to directly compare the runtime among them. Just to get a sense about the run time. Following table summarises the run time.

Data	**Mapinsights bamqc**	**Samtools stats**	**Alfred qc**	**Qualimap 2 bamqc**	**Picard (CollectRawWgsMetrics)**
NA12878 whole genome (~15X)	35m : 26s	19m : 56s	30m : 51s	51m : 2s<br>NOTE :<br>--java-mem-size=6G	58m : 08s

All tools are run on Intel(R) Core(TM) i7-9700 CPU @ 3.00GHz with 1 core and in default mode except qualimap2. Qualimap2 throws a memory error for --java-mem-size <=4GB. So we run it with --java-mem-size=6G. Samtools-stats took least time to complete the analysis because it included less number of features compared to other tools. Among the rest of the tools (except samtool stats) Mapinsights-bamqc modules show comparable or less run time, although it includes few novel features that are computationally intensive.

Processing of open source data

For Nextera data FASTQ files were downloaded from Genome in a bottle consortium (See Supplementary Table S1 for more details). The reads are generated on two different lanes of a single flow-cell using two different indexes. The FASTQ files contain ~168.4 million reads of length 100bp. Lane and index wise processing was done following GATK best practices protocols. Downloaded reads are aligned against hg19 decoy reference sequences using BWA mem. Sequence alignment map (SAM) file generated after alignment was sorted based on chromosomal coordinates and converted to BAM file using PICARD. Afterwards, the BAM file fed to the PICARD tool for marking the optical duplicates followed by indel realignment and base quality recalibration using GATK. Lane and index wise recalibrated BAM files were merged to generate the final aligned file.

For TruSeq data, FASTQ files were not accessible from GIAB resources. Two BAM files namely NIST-hg001-7001-ready.bam and NIST-hg001-7001-b-ready.bam were available. NIST-hg001-7001-ready.bam was taken for analysis. Samtools was used to sort the BAM file according to read-id and converted to paired FASTQ files using BEDtools (65). Resultant FASTQ contains reads of various lengths and maximum read length was 150bp. Out of ~53 million reads present in the FASTQ files ~37 million reads (69.8%) are taken based on read length >= 148bp for further processing. This data was sequenced on two different lanes of a single flow-cell. The selected reads are then lane wise separated and processed following protocol mentioned above. Finally, a BAM file was generated by merging the final alignment file from both the lanes. The mean depth of coverage is ~30X for TruSeq whereas ~146X for Nextera. We have downsampled the Nextera data to ~30X for better comparison between both the data sets. Similarly. For lung carcinoma data we only extract reads having read length of 100 from FASTQ files and follow the best practice protocol for further processing. See supplementary Data 5 for detailed Information pertains to data downloads.

References

65. Quinlan AR, Hall IM. BEDTools: a flexible suite of utilities for comparing genomic features. Bioinformatics 2010; 26:841–842

Supplementary Results

Mapinsights-bamqc feature description with respect to HG01402 sample:

We have observed a sudden rise in substitution rate in cycle number 62 in the overall per cycle profile. As the *bamqc* module provides detailing in paired-end orientation fashion so we are able to enquire further with respect to cycle specific error. We found that the bias occurs only in the second-in-pair and in both the strands (Supplementary Figure S3A). C>A/G>T and A>C/T>G are found to be the major substitutions as observed in the overall substitution profile. On further investigation, we observed C>A/G>T substitution has strand specificity. C>A occurs more in the forward strand and G>T in the reverse strand whereas A>C/T>G occurs almost equally in both the strands (Supplementary Figure S3B). Further from Supplementary Figure S3C, it is evident that read2 contains more errors compared to read1.

Profile of exome sequencing data downloaded from 1000G:

Profile of substitution categories: The substitution pairs like C>T/G>A, A>T/T>A etc have shown similar trends in percentage change with the exception in G>T and C>A where the deviation is sharp. Like whole genome HiSeq data here also A>C and G>T suffer the most with respect to low base quality where more than 50% of such changes have base quality <=10 in almost all 30 samples (Figure 34-middle panel).

Per cycle profile: The average substitution rate is around 1.27 in all cycles in all 30 samples and the range is between o.53 to 33.3. In general, the rate is higher in the first cycle and in the last five cycles. There is a sharp drop in quality observed in the first and last cycles, however even if the substitution rate is higher in the last few cycles (except last one), an increase in base quality is also observed (Figure 6A) indicating error incorporation before sequencing most likely due to end-repair artefacts. C>T/G>A and A>G/T>C are the major nucleotide changes across cycles and decrease at the end (Figure 6A-lower panel) whereas A>C/T>G occur more at the end of the reads, showing a reverse pattern. A>T/T>A substitutions are higher in first base which is unusual and likely artifacts.

Read mismatch content: In 30 samples the average % of reads having one mismatch is 14.68 with range from 11.98 to 18.97(Supplementary Figure S7) and the percentage decreases sharply with increased mismatches.

Exonwise gene depth of coverage profile: Depth status of *BRCA1* gene is used to explain the functionality and usefulness of Mapinsight's genedepth module on two samples i.e HG00114 and HG02155 (Figure 6D). In the exome target bed file downloaded from 1000G has 21 exons in it for BRCA1 gene. The mean depth of coverage (DCOV) for BRCA1 is around 122X in sample HG00114, whereas it is 88X in HG02155, however DCOV varies across different exons and it ranges from 0 to 228X in HG02155 and the range is 20 to 210X in HG00114.

Profile of NA12878 exome sequencing data (Truseq Vs Nextera):

Profile of substitution categories: It is observed that A>G/T>C (11.4%/11.6%) transversions are the highest among other substitution categories in Nextera data whereas C>A(13.7%) transitions are predominant in TruSeq data which is more than 2 fold higher compared to G>T(5.7%) substitutions indication error (Figure 4C-upper panel). Importantly the quality score of C>A changes are mostly (70%) > QV 20, that means good quality bases suggesting the errors might have occurred before sequencing possibly during library preparation. Like the other datasets analyzed here also we observed the same pattern, i.e, more than 70% A>C and T>G changes have quality < 10 and an abundance of C_C (30.49%) and G_G (29.93%) context (Figure 4C-lower panel). Additionally, two other contextual changes were observed in higher frequency i.e GAG>GGG and CTC>CCC in nextera data.

Per cycle profile: In Nextera data (Figure 6B), the average substitution rate per cycle is ~1.5 and a significantly high substitution rate is observed in cycle number 3,4 and the last cycles with substitution rate 3.5, 2.1 and 5.5 respectively. Whereas cycle number 95 is severely affected in TruSeq data with a substitution rate ~15.2, however the other cycles are not that much affected and the substitution rates are relatively low, mostly similar to other nearby cycles. Additionally, C>A substitutions are predominant in each cycle except cycle number 95 in Truseq data (Figure 6C).

Exon-wise gene depth of coverage profile: We have run the genedepth module on both Nextera and TruSeq data sets for *CDKN2A* gene. *CDKN2A* was found to be associated with different diseases including multiple cancers. The number of exons (n=4) and their coordinates are the same in both TruSeq and Nextera captures. The bam files used in this analysis have ~30X mean depth of coverage for both the data. In Nextera data the mean DCOV of *CDKN2A* gene is 19.38X whereas it is 24.55X in TruSeq data (Figure 6E).

Profile of fresh frozen and & FFPE exomes:

Profile of substitution categories: A>G and T>C are the dominating changes (> 13.7%) observed in fresh frozen tumor and normal samples in both the patients whereas C>T and G>A substitutions are found to be major changes (>13.9%) in both the FFPE data reflecting the know error signal associated with FFPE (Figure 4D).

Per cycle profile: Substitution rate is higher at both end cycles in case of FFPE data compared to fresh frozen data. In T3-FFPE data cycle number 94 has a substitution rate of 4.7% that is 2 fold higher compared to nearby cycles indicating bias and a drop of quality also observed in the same cycle (Supplementary Figure S8).

Supplementary Figures

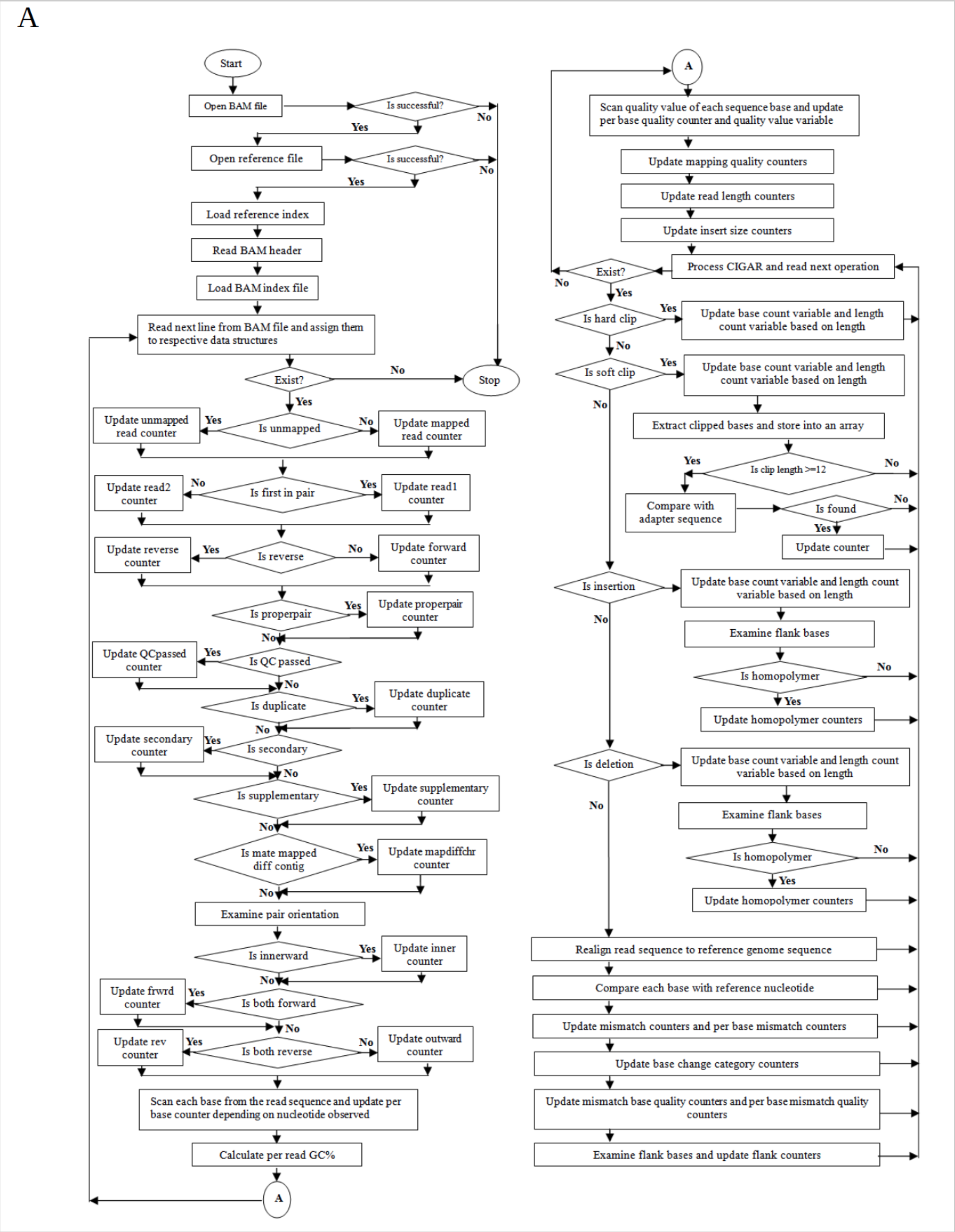

Figure S1. Flowchart of *bamqc* module of Mapinsights.

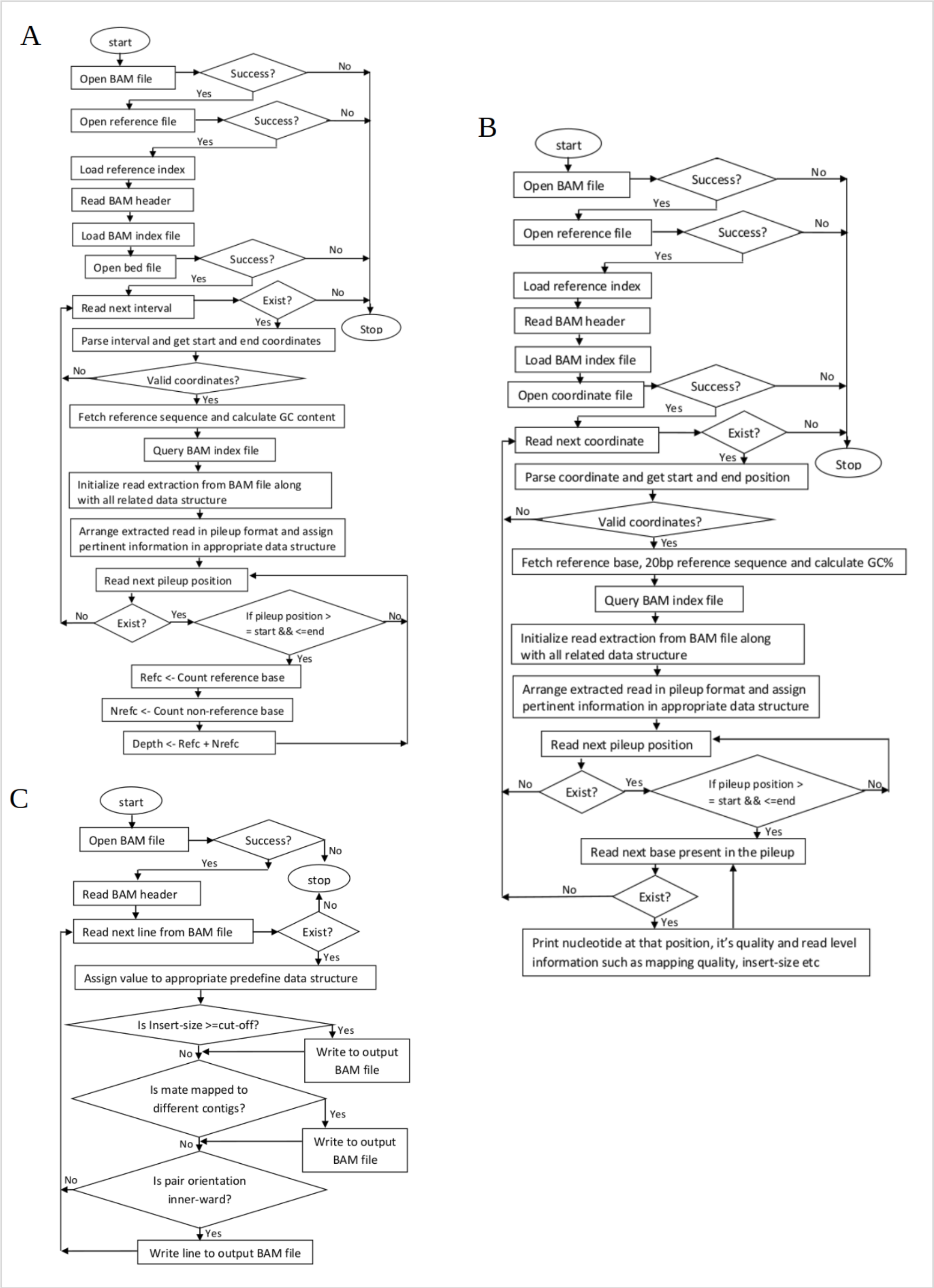

Figure S2. **(A)** Flowchart of *genedepth* module. **(B)** Flowchart of *siteinfo* module **(C)** Flowchart of *jumpreads* module.

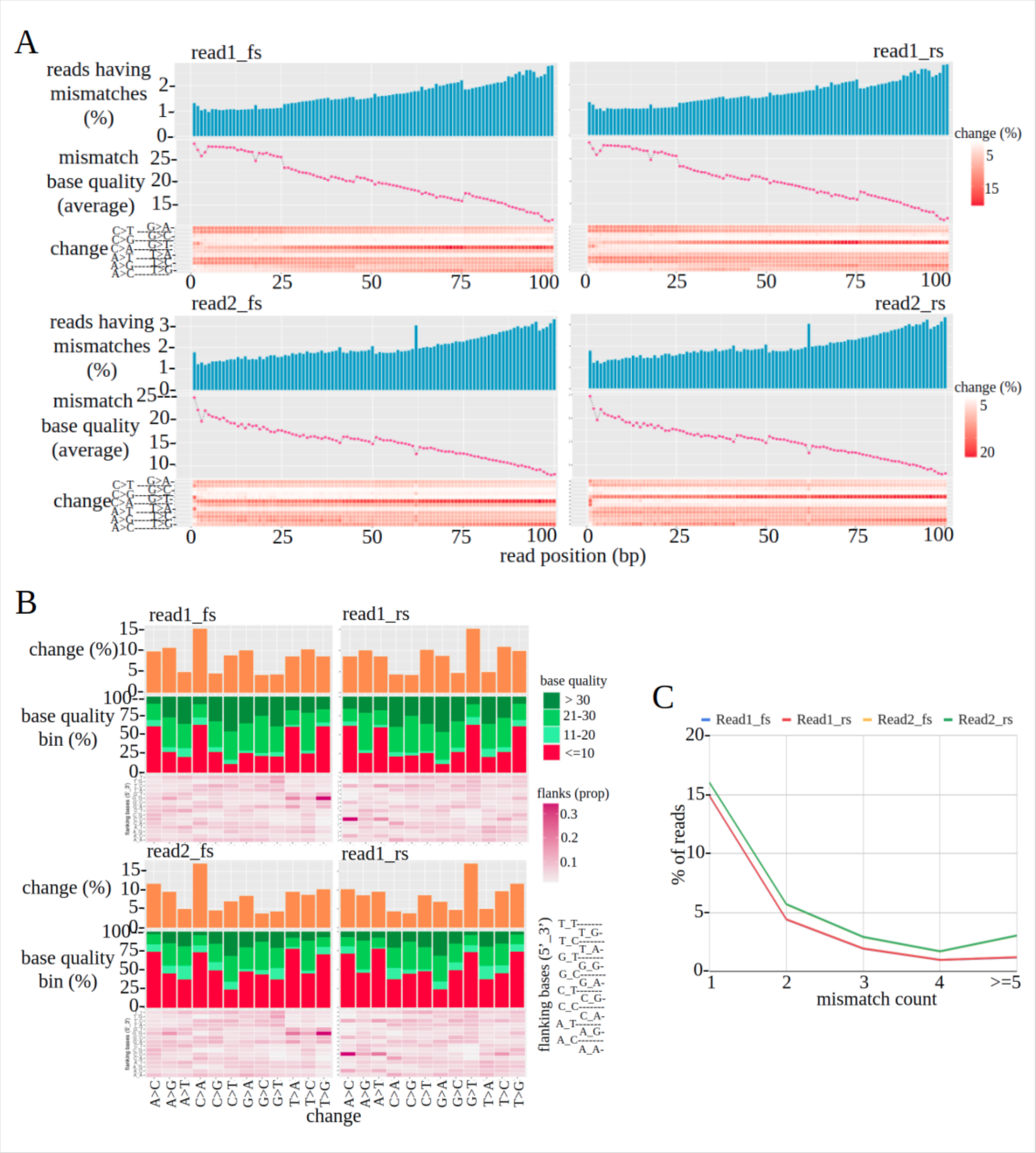

Figure S3. **(A)** *Per-cycle-profile* of HG01402 sequence data in paired-end orientation manner **(B)** *Profile-of-substitution-categories* of HG01402 sequence data in paired-end orientation manner **(C)** *Read-mismatch-content* profile in paired-end orientation manner. Higher mismatch observed for read 2. Here, *read1_fs* : first in pair and forward strand; *read1_rs* : first in pair and reverse strand; *read2_fs* : second in pair and forward strand; *read2_fs* : second in pair and reverse strand.

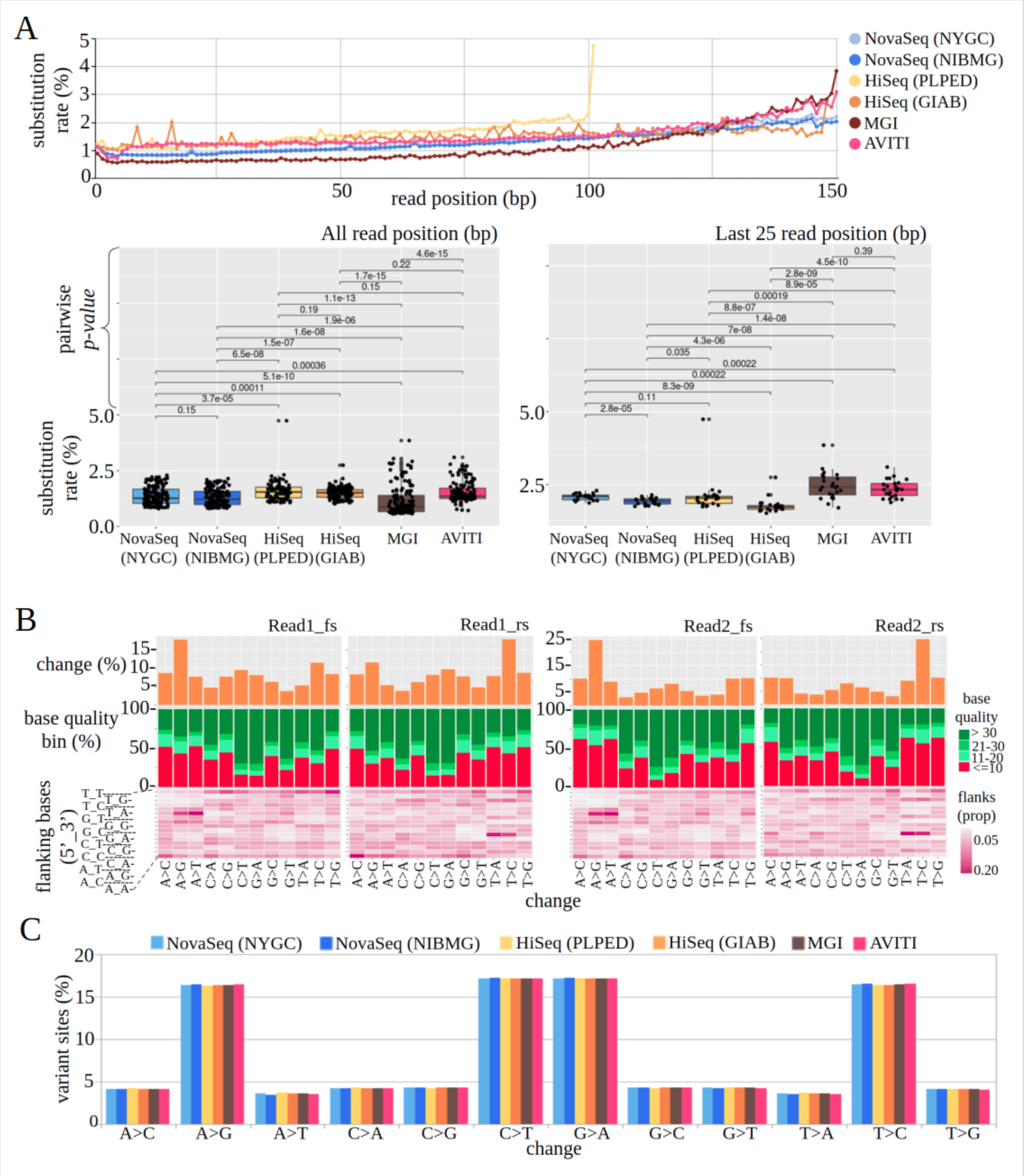

Figure S4. **(A)** Combined representation of per-cycle substitution rate across various sequencing platforms (upper) and pairwise comparison (wilcoxon test) of substitution rate between them (below). **(B)** *profile-of-substitution-categories* of MGI data in paired-end orientation manner. Here, *read1_fs* : first in pair and forward strand; *read1_rs* : first in pair and reverse strand; *read2_fs* : second in pair and forward strand; *read2_fs* : second in pair and reverse strand. **(C)** Distribution of substitution categories in pass filter variant calls generated from NovaSeq, HiSeq, MGI and AVITI sequencing data.

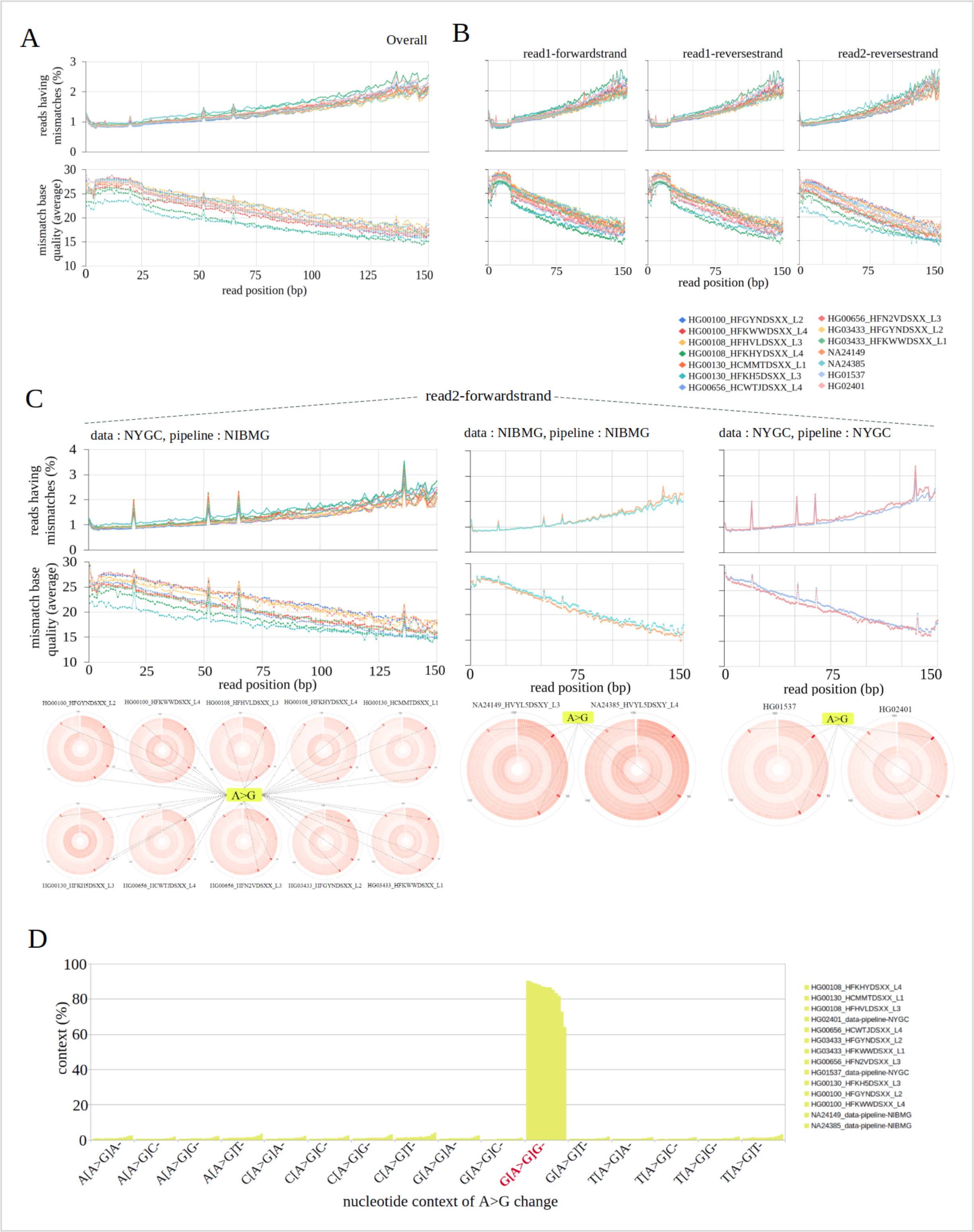

Figure S5. To validate the cycle specific error pattern observed in NA12878 sample sequenced in two different labs (NYGC, NIBMG), we have used additional WGS datasets generated from NYGC and NIBMG using NovaSeq sequencer. Mapinsights-*bamqc* module was run on these additional data sets. **(A)** per cycle profile of substitution rate and average mismatch base quality of additional data in overall reads. Individual samples were marked in different colored lines. **(B)** per cycle profile of substitution rate and average mismatch base quality of additional data in paired-end orientation manner (except read2-forward strand reads). **(C)** Per cycle profile of read2-forward strand reads in additional data sets (left). Profiles on additional data were further subdivided based on the sequencing center (right). % of occurence of different substitution categories per cycle (bottom).

(D) distribution of 16 different nucleotide contexts of A>G substitution found in 4 affected sequencing cycles in additional NovaSeq data.

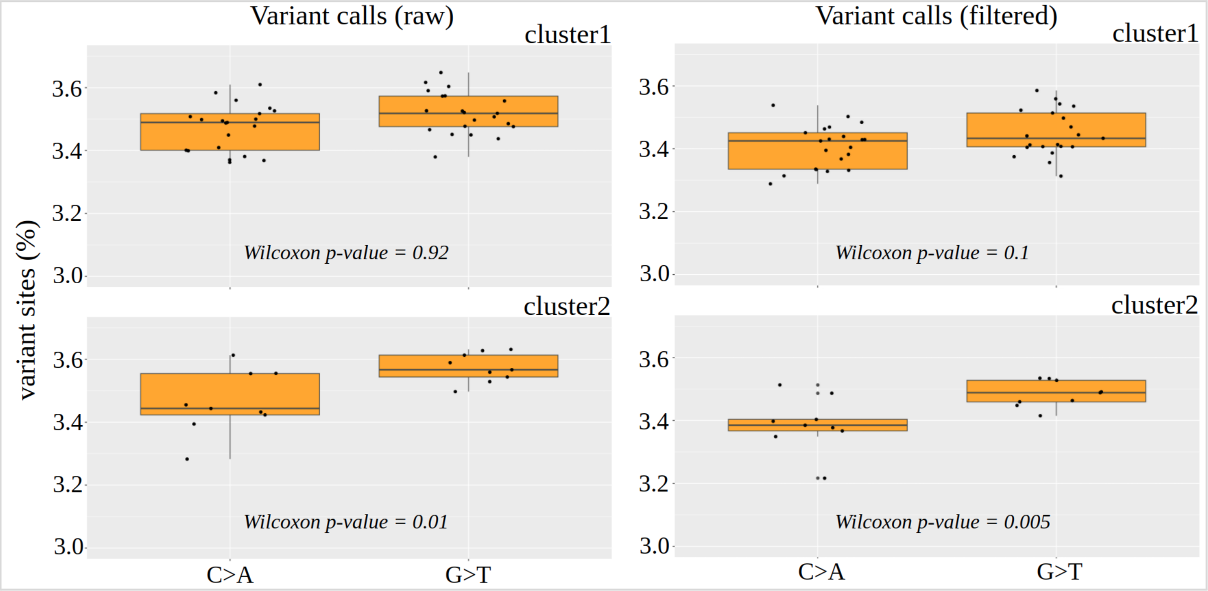

Figure S6. Percentage of C>A and G>T variants (both raw (left) and filtered (right)) between two clusters; cluster1 : variant calls from 21 exomes and cluster2 : variant calls from 9 exomes. A significant deviation between C>A and G>T is observed in cluster2 with *p-value = 0.01* in raw and *p-value = 0.005* in filtered variant calls across 9 exomes.

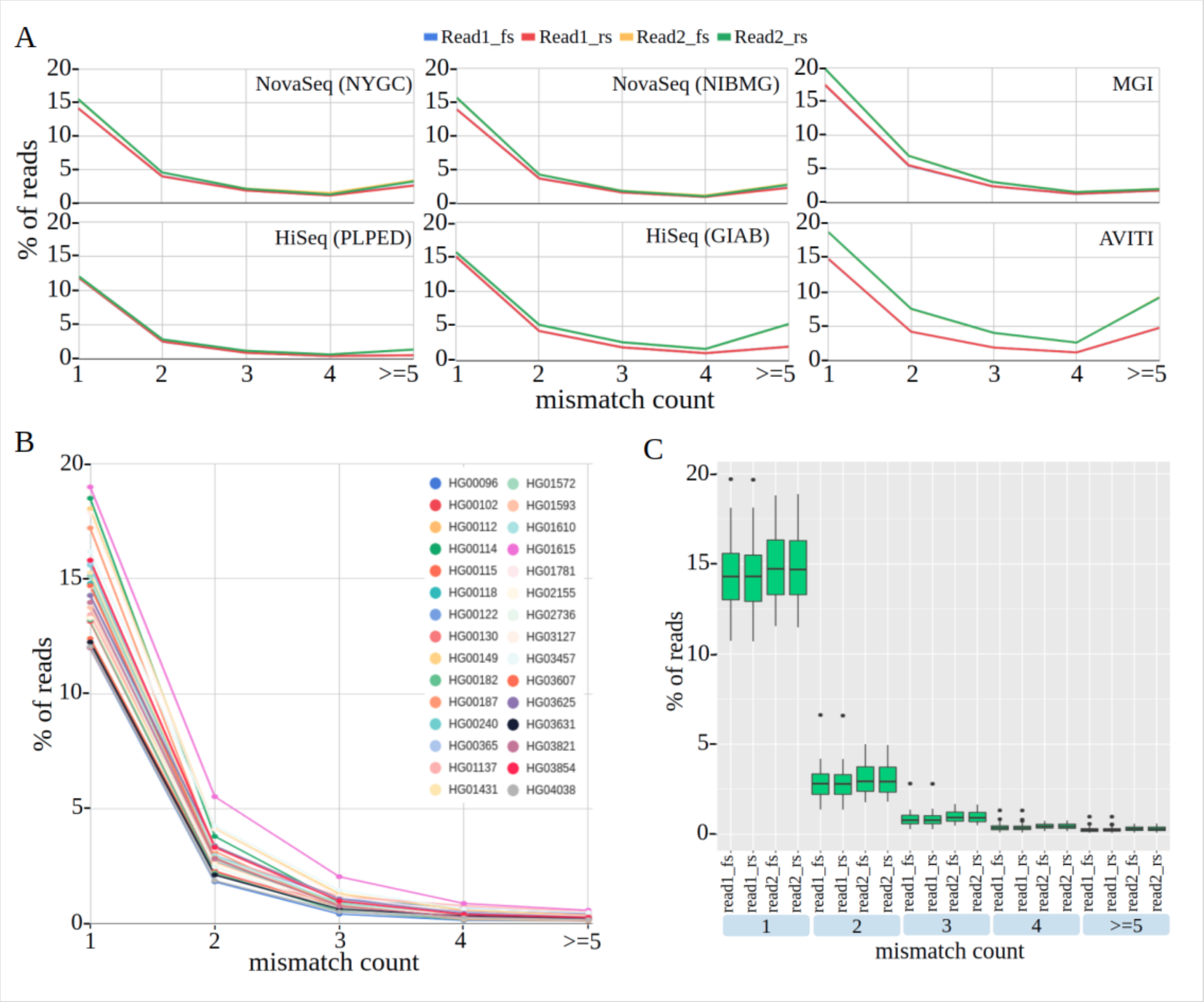

Figure S7. *Read-mismatch-content* profile of NA12878 sample sequence in various sequencing platforms (HiSeq, NovaSeq, MGI and AVITI) and also of 30 exome datasets sequenced using HiSeq in overall and paired-end orientation manner. In each data set, read2 contains more mismatches compared to read1.

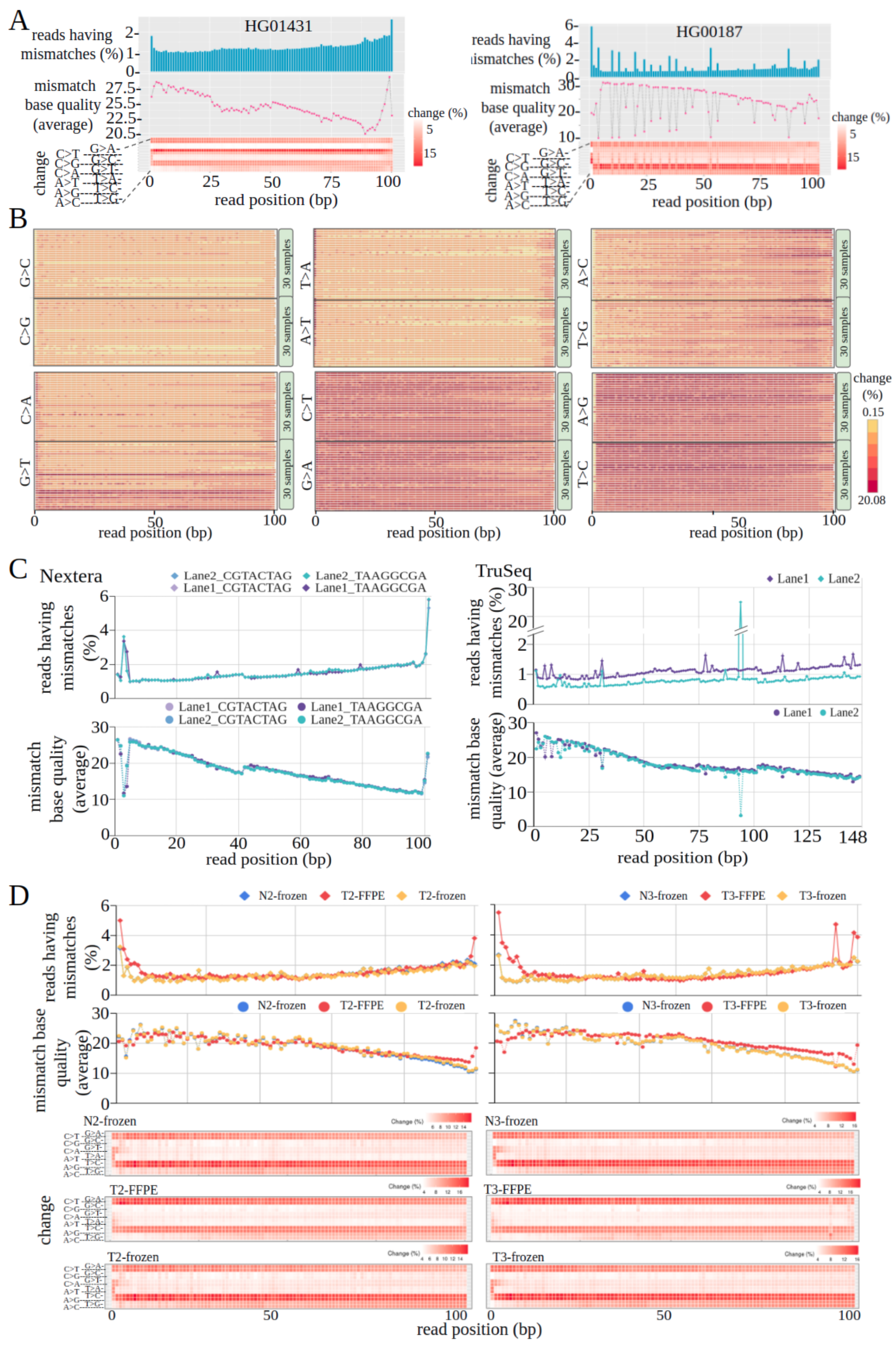
A
HG01431
reads having mismatches (%)
mismatch base quality (average)
change (%)
change
read position (bp)
HG00187
B
G>C
C>G
T>A
A>T
A>C
T>G
C>A
G>T
C>T
G>A
A>G
T>C
30 samples
change (%)
0.15
20.08
read position (bp)
C
Nextera
Lane2_CGTACTAG
Lane2_TAAGGCGA
Lane1_CGTACTAG
Lane1_TAAGGCGA
TruSeq
Lane1
Lane2
reads having mismatches (%)
mismatch base quality (average)
read position (bp)
D
N2-frozen
T2-FFPE
T2-frozen
N3-frozen
T3-FFPE
T3-frozen
reads having mismatches (%)
mismatch base quality (average)
change
read position (bp)

Figure S8. (A) Contrasting example of two samples (HG01431 and HG00187) with respect to cycle bias belonging to 30 exome sequencing data. **(B)** Substitution type wise per cycle substitution profile of 30 exome sequencing data. **(C)** Lane-wise per cycle profile of Nextera and TruSeq data. **(D)** per cycle profile of three data types (formalin-fixed paraffin-embedded tumor tissue, fresh frozen normal and tumor tissue data) of two lung adenocarcinoma patients.

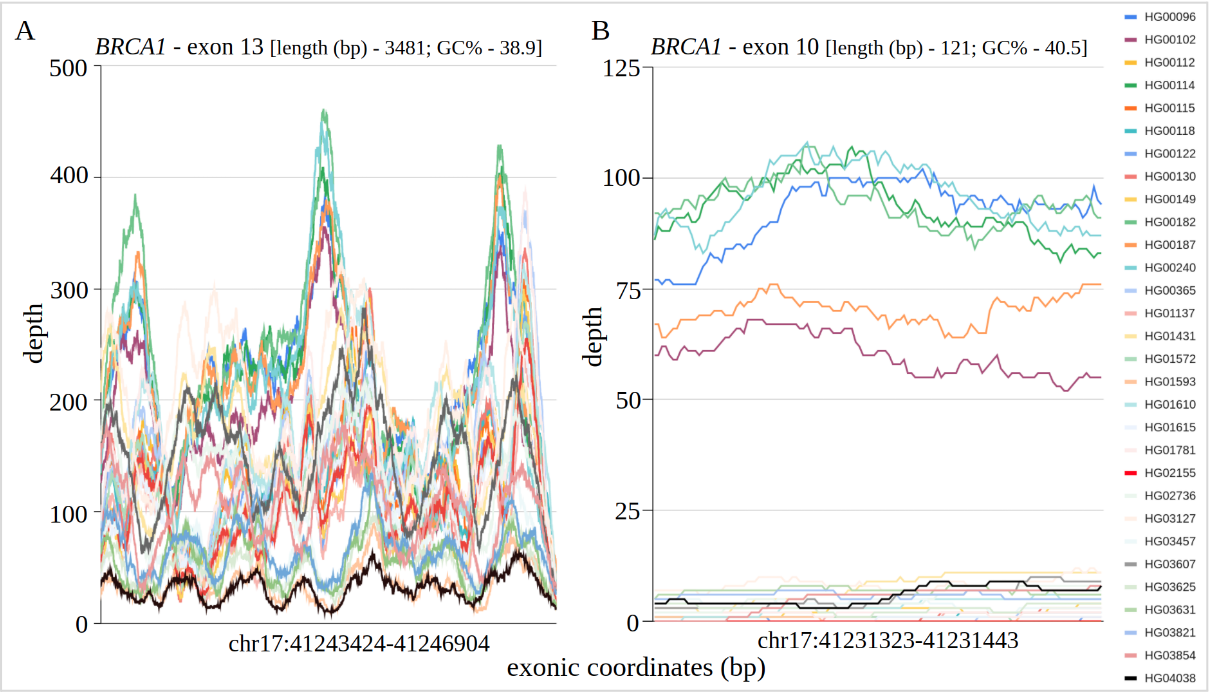

Figure S9. (A, B) coverage plot of *BRAC1*- exon13 and *BRCA1*-exon10 in 30 exome sequencing data.

Supplementary Tables

Table S1 : A comparative view of different QC tools with respect to different features included in the *bamqc* module of Mapinsights. As can be observed, the bamqc module has capabilities similar to state-of-the-art existing tools, together with some additional unique features that provide new perception of data which are instrumental and useful with respect to QC of aligned data.
NOTE : Yes# : Did not provide the feature directly but can be calculated from some other logs; Combined : Combined logs are provided. Since, MultiQC only generate combined reports based on results and log files generated by other bioinformatics tools, it is not included in the table.

Features	Mapinsights bamqc	Samtools stats	Qualimap 2	FastQC	Alfred qc	Picard
Mapped-reads	Yes	Yes	Yes	No	Yes	Yes
Mapped-read1 & read2	Yes	Yes	Yes	No	Yes	Yes
Mapped-forward & reverse	Yes	No	No	No	Yes	No
Mapped-pair	Yes	Yes	Yes	No	Yes	Yes
Mapped-properpair	Yes	Yes	No	No	Yes	Yes
Secondary & Supplementary alignments	Yes	Yes	No	No	Yes	Yes
QC-failed	Yes	Yes	No	No	Yes	Yes
Strand-ratio (F:R)	Yes	No	No	No	Yes	Yes
Softclip-events & basecounts	Yes	No	No	No	Partial	No
Hardclip-events & basecounts	Yes	No	No	No	Partial	No
Illumina-Adapter & PCR-primer	Yes	No	No	Yes	No	Yes
Nextera-Transposase-Sequence	Yes	No	No	Yes	No	No
A : T : G : C : N	Yes	No	Yes	No	No	No
GC %	Yes	Yes	Yes	Yes	Yes	Yes
Single nucleotide mismatch counts	Yes	Yes	Yes	No	Yes	No
INDEL events	Yes	Yes#	Yes	No	Yes	No
INDEL basecount	Yes	No	No	No	No	No
INDEL rate	Yes	Yes#	Yes#	No	Yes	Yes
Homopolymer INDEL	Yes	No	Yes	No	Yes	No
Mismatch rate	Yes	Yes	Yes	No	Yes	Yes
Pair mapped to different contig	Yes	Yes	No	No	Yes	No
Insert Size>=1K	Yes	Yes#	Yes#	No	Yes#	Yes#
Mapped-pair forward:reverse:outward:innerward	Yes : Yes :Yes :Yes	Combined : Yes : Yes	No : No : No :No	No : No : No :No	No : No : No :No	No : No : No :No
Mean/Median depth of coverages	Yes	Yes	Yes	No	Yes	Yes
Mean/Median insert size	Yes	Yes	Yes	No	Yes	Yes
Mean/Median base quality	Yes	Yes	No	No	No	Yes
Mean/Median mapping quality	Yes	No	Yes	No	Yes	No
Mean/Median read length	Yes	Yes	Yes	Yes	Yes	Yes
DuplicateMarked rate	Yes	Yes	Yes	Yes	Yes	Yes
Per base nucleotide content	Yes	Yes	Yes	Yes	Yes	Yes
GC content distribution	Yes	Yes	Yes	Yes	Yes	Yes
Insert Size distribution	Yes	Yes	Yes	No	Yes	Yes
Base quality distribution	Yes	Yes	No	No	No	Yes
Mean quality per cycle	Yes	Yes	Yes	Yes	Yes	Yes
Mapping quality distribution	Yes	No	Yes	No	Yes	No

Per read mismatch rate	Yes	No	No	No	No	No
Distribution of substitution categories	Yes	No	No	No	No	Yes
Quality value distribution per substitution categories	Yes	No	No	No	No	No
Distribution of flanking bases per substitution categories	Yes	No	No	No	No	Yes
Per cycle substitution rate	Yes	No	No	No	No	No
Per cycle average quality of mismatch base	Yes	No	No	No	No	No
Proportion of substitution categories per cycle	Yes	No	No	No	No	No
Length distribution of insertion & deletion	Yes	Yes	No	No	Yes	No
Length distribution of clipping events	Yes	No	No	No	No	No

Table S2 : Download sources of various data files

Source	Sample	Link	Data type downloaded	Sequencing type	Sequencer	Remarks
1000G	HG01402	ftp://ftp.sra.ebi.ac.uk/vol1/fastq/ERR251/ERR251180/ERR251180_1.fastq.gz	fastq	whole genome	HiSeq	
		ftp://ftp.sra.ebi.ac.uk/vol1/fastq/ERR251/ERR251180/ERR251180_2.fastq.gz				
		ftp://ftp.sra.ebi.ac.uk/vol1/fastq/ERR251/ERR251885/ERR251885_1.fastq.gz				
		ftp://ftp.sra.ebi.ac.uk/vol1/fastq/ERR251/ERR251885/ERR251885_2.fastq.gz				
1000G-Illumina Platinum pedigree	NA12878	ftp://ftp.sra.ebi.ac.uk/vol1/fastq/ERR194/ERR194147/ERR194147_1.fastq.gz				
		ftp://ftp.sra.ebi.ac.uk/vol1/fastq/ERR194/ERR194147/ERR194147_2.fastq.gz				
Ginome in a bottle		ftp-trace.ncbi.nlm.nih.gov/ReferenceSamples/giab/data/NA12878/NIST_NA12878_HG001_HiSeq_300x/140127_D00360_0012_BH8GVUADXX/Project_RM8398/				
1000G-New York Genome Center		ftp.sra.ebi.ac.uk/vol1/fastq/ERR323/004/ERR3239334/ERR3239334_1.fastq.gz			NovaSeq	
		ftp.sra.ebi.ac.uk/vol1/fastq/ERR323/004/ERR3239334/ERR3239334_2.fastq.gz				
	HG01537	ftp.sra.ebi.ac.uk/vol1/run/ERR324/ERR3241939/HG01537.final.cram	cram			
	HG02401	ftp.sra.ebi.ac.uk/vol1/run/ERR324/ERR3242285/HG02401.final.cram				
	HG00100	ftp.sra.ebi.ac.uk/vol1/run/ERR423/ERR4231860/HG00100_GTGAATAT-TCTCATTC_HFGYNDSXX_L002_001.R1.fastq.gz	fastq			
		ftp.sra.ebi.ac.uk/vol1/run/ERR423/ERR4231860/HG00100_GTGAATAT-TCTCATTC_HFGYNDSXX_L002_001.R2.fastq.gz				
		ftp.sra.ebi.ac.uk/vol1/run/ERR423/ERR4231852/HG00100_GTGAATAT-TCTCATTC_HFKWWDSXX_L004_001.R1.fastq.gz				
		ftp.sra.ebi.ac.uk/vol1/run/ERR423/ERR4231852/HG00100_GTGAATAT-TCTCATTC_HFKWWDSXX_L004_001.R2.fastq.gz				
	HG00108	ftp.sra.ebi.ac.uk/vol1/run/ERR398/ERR3986407/HG00108_ACACTAAG-ATCCATAT_HFHVLDSXX_L003_001.R1.fastq.gz				
		ftp.sra.ebi.ac.uk/vol1/run/ERR398/ERR3986407/HG00108_ACACTAAG-ATCCATAT_HFHVLDSXX_L003_001.R2.fastq.gz				
		ftp.sra.ebi.ac.uk/vol1/run/ERR398/ERR3986401/HG00108_ACACTAAG-ATCCATAT_HFKHYDSXX_L004_001.R1.fastq.gz				
		ftp.sra.ebi.ac.uk/vol1/run/ERR398/ERR3986401/HG00108_ACACTAAG-ATCCATAT_HFKHYDSXX_L004_001.R2.fastq.gz				
	HG00130	ftp.sra.ebi.ac.uk/vol1/run/ERR356/ERR3568897/HG00130_GTGTCGGA-GCTTGCGC_HCMMTDSXX_L001_001.R1.fastq.gz				
		ftp.sra.ebi.ac.uk/vol1/run/ERR356/ERR3568897/HG00130_GTGTCGGA-GCTTGCGC_HCMMTDSXX_L001_001.R2.fastq.gz				
		ftp.sra.ebi.ac.uk/vol1/run/ERR356/ERR3568903/HG00130_GTGTCGGA-GCTTGCGC_HFKH5DSXX_L003_001.R1.fastq.gz				
		ftp.sra.ebi.ac.uk/vol1/run/ERR356/ERR3568903/HG00130_GTGTCGGA-GCTTGCGC_HFKH5DSXX_L003_001.R2.fastq.gz				
	HG00656	ftp.sra.ebi.ac.uk/vol1/run/ERR423/ERR4231901/HG00656_TCGTAGTG-AGACTTGG_HCWTJDSXX_L004_001.R1.fastq.gz				
		ftp.sra.ebi.ac.uk/vol1/run/ERR423/ERR4231901/HG00656_TCGTAGTG-AGACTTGG_HCWTJDSXX_L004_001.R2.fastq.gz				
		ftp.sra.ebi.ac.uk/vol1/run/ERR423/ERR4231908/HG00656_TCGTAGTG-AGACTTGG_HFN2VDSXX_L003_001.R1.fastq.gz				
		ftp.sra.ebi.ac.uk/vol1/run/ERR423/ERR4231908/HG00656_TCGTAGTG-AGACTTGG_HFN2VDSXX_L003_001.R2.fastq.gz				
	HG03433	ftp.sra.ebi.ac.uk/vol1/run/ERR423/ERR4231872/HG03433_AACAGGTT-ATACCAAG_HFGYNDSXX_L002_001.R1.fastq.gz				
		ftp.sra.ebi.ac.uk/vol1/run/ERR423/ERR4231872/HG03433_AACAGGTT-ATACCAAG_HFGYNDSXX_L002_001.R2.fastq.gz				
		ftp.sra.ebi.ac.uk/vol1/run/ERR423/ERR4231869/HG03433_AACAGGTT-ATACCAAG_HFKWWDSXX_L001_001.R1.fastq.gz				
		ftp.sra.ebi.ac.uk/vol1/run/ERR423/ERR4231869/HG03433_AACAGGTT-ATACCAAG_HFKWWDSXX_L001_001.R2.fastq.gz				
1000G	HG00096	ftp.1000genomes.ebi.ac.uk/vol1/ftp/phase3/data/HG00096/exome_alignment/HG00096.mapped.ILLUMINA.bwa.GBR.exome.20120522.bam	bam	Exome	HiSeq	
	HG00102	ftp.1000genomes.ebi.ac.uk/vol1/ftp/phase3/data/HG00102/exome_alignment/HG00102.mapped.ILLUMINA.bwa.GBR.exome.20121211.bam				
	HG00112	ftp.1000genomes.ebi.ac.uk/vol1/ftp/phase3/data/HG00112/exome_alignment/HG00112.mapped.ILLUMINA.bwa.GBR.exome.20130415.bam				
	HG00114	ftp.1000genomes.ebi.ac.uk/vol1/ftp/phase3/data/HG00114/exome_alignment/HG00114.mapped.ILLUMINA.bwa.GBR.exome.20120522.bam				
	HG00115	ftp.1000genomes.ebi.ac.uk/vol1/ftp/phase3/data/HG00115/exome_alignment/HG00115.mapped.ILLUMINA.bwa.GBR.exome.20130415.bam				
	HG00118	ftp.1000genomes.ebi.ac.uk/vol1/ftp/phase3/data/HG00118/exome_alignment/HG00118.mapped.ILLUMINA.bwa.GBR.exome.20130415.bam				
	HG00122	ftp.1000genomes.ebi.ac.uk/vol1/ftp/phase3/data/HG00122/exome_alignment/HG00122.mapped.ILLUMINA.bwa.GBR.exome.20130415.bam				
	HG00130	ftp.1000genomes.ebi.ac.uk/vol1/ftp/phase3/data/HG00130/exome_alignment/HG00130.mapped.ILLUMINA.bwa.GBR.exome.20130415.bam				
	HG00149	ftp.1000genomes.ebi.ac.uk/vol1/ftp/phase3/data/HG00149/exome_alignment/HG00149.mapped.ILLUMINA.bwa.GBR.exome.20130415.bam				
	HG00182	ftp.1000genomes.ebi.ac.uk/vol1/ftp/phase3/data/HG00182/exome_alignment/HG00182.mapped.ILLUMINA.bwa.FIN.exome.20120522.bam				
	HG00187	ftp.1000genomes.ebi.ac.uk/vol1/ftp/phase3/data/HG00187/exome_alignment/HG00187.mapped.ILLUMINA.bwa.FIN.exome.20120522.bam				
	HG00240	ftp.1000genomes.ebi.ac.uk/vol1/ftp/phase3/data/HG00240/exome_alignment/HG00240.mapped.ILLUMINA.bwa.GBR.exome.20121211.bam				
	HG00365	ftp.1000genomes.ebi.ac.uk/vol1/ftp/phase3/data/HG00365/exome_alignment/HG00365.mapped.ILLUMINA.bwa.FIN.exome.20130415.bam				
	HG01137	ftp.1000genomes.ebi.ac.uk/vol1/ftp/phase3/data/HG01137/exome_alignment/HG01137.mapped.ILLUMINA.bwa.CLM.exome.20130415.bam				
	HG01431	ftp.1000genomes.ebi.ac.uk/vol1/ftp/phase3/data/HG01431/exome_alignment/HG01431.mapped.ILLUMINA.bwa.CLM.exome.20130415.bam				
	HG01572	ftp.1000genomes.ebi.ac.uk/vol1/ftp/phase3/data/HG01572/exome_alignment/HG01572.mapped.ILLUMINA.bwa.PEL.exome.20130415.bam				
	HG01593	ftp.1000genomes.ebi.ac.uk/vol1/ftp/phase3/data/HG01593/exome_alignment/HG01593.mapped.ILLUMINA.bwa.PJL.exome.20130415.bam				
	HG01610	ftp.1000genomes.ebi.ac.uk/vol1/ftp/phase3/data/HG01610/exome_alignment/HG01610.mapped.ILLUMINA.bwa.IBS.exome.20130415.bam				
	HG01615	ftp.1000genomes.ebi.ac.uk/vol1/ftp/phase3/data/HG01615/exome_alignment/HG01615.mapped.ILLUMINA.bwa.IBS.exome.20130415.bam				
	HG01781	ftp.1000genomes.ebi.ac.uk/vol1/ftp/phase3/data/HG01781/exome_alignment/HG01781.mapped.ILLUMINA.bwa.IBS.exome.20130415.bam				
	HG02155	ftp.1000genomes.ebi.ac.uk/vol1/ftp/phase3/data/HG02155/exome_alignment/HG02155.mapped.ILLUMINA.bwa.CDX.exome.20130415.bam				
	HG02736	ftp.1000genomes.ebi.ac.uk/vol1/ftp/phase3/data/HG02736/exome_alignment/HG02736.mapped.ILLUMINA.bwa.PJL.exome.20130415.bam				
	HG03127	ftp.1000genomes.ebi.ac.uk/vol1/ftp/phase3/data/HG03127/exome_alignment/HG03127.mapped.ILLUMINA.bwa.ESN.exome.20130415.bam				
	HG03457	ftp.1000genomes.ebi.ac.uk/vol1/ftp/phase3/data/HG03457/exome_alignment/HG03457.mapped.ILLUMINA.bwa.MSL.exome.20130415.bam				
	HG03607	ftp.1000genomes.ebi.ac.uk/vol1/ftp/phase3/data/HG03607/exome_alignment/HG03607.mapped.ILLUMINA.bwa.BEB.exome.20130415.bam				
	HG03625	ftp.1000genomes.ebi.ac.uk/vol1/ftp/phase3/data/HG03625/exome_alignment/HG03625.mapped.ILLUMINA.bwa.PJL.exome.20130415.bam				
	HG03631	ftp.1000genomes.ebi.ac.uk/vol1/ftp/phase3/data/HG03631/exome_alignment/HG03631.mapped.ILLUMINA.bwa.PJL.exome.20130415.bam				
	HG03821	ftp.1000genomes.ebi.ac.uk/vol1/ftp/phase3/data/HG03821/exome_alignment/HG03821.mapped.ILLUMINA.bwa.BEB.exome.20130415.bam				
	HG03854	ftp.1000genomes.ebi.ac.uk/vol1/ftp/phase3/data/HG03854/exome_alignment/HG03854.mapped.ILLUMINA.bwa.STU.exome.20130415.bam				
	HG04038	ftp.1000genomes.ebi.ac.uk/vol1/ftp/phase3/data/HG04038/exome_alignment/HG04038.mapped.ILLUMINA.bwa.STU.exome.20130415.bam				
Ginome in a bottle	NA12878	ftp://ftp-trace.ncbi.nih.gov/ReferenceSamples/giab/data/NA12878/				TruSeq
			fastq			Nextera
PRJNA301548: SRP065941	N2_frozen	https://www.ncbi.nlm.nih.gov/Traces/study/?acc=SRP065941&o=acc_s%3Aa				
	T2_FFPE					
	T2_frozen					
	N3_frozen					
	T3_FFPE					
	T3_frozen					
PRJEB25641	NA12878		fastq	whole genome	MGI-BGISEQ-500	
s3://gencove-element-paper-2022, PRJNA909799	NA12878		bam, vcf	whole genome	Element AVITI	

Table S3 : Run time of Mapinsights modules

Modules of Mapinsights	Whole genome (~15X) [minute : second]	Exome (~30X) [minute : second]	Remarks
bamqc	35m : 26.602s	3m : 11.481s	
genedepth	0m : 16.255s	0m : 16.340s	For BRCA1 gene having 21 exons
siteinfo	0m : 0.613s	0m : 0.597s	For a single site
jumpreads	14m : 9.395s	0m : 58.461s	

Table S4 : Exon-wise depth status of *HRAS* gene in a 1000G project dataset (HG01402 sample)

No of exones	4
Target bed size (bp)	1020
Mean depth of coverage	5.58X

Exon no	Exon1	Exon2	Exon3	Exon4
Genomic coordinates	chr11:532575-532815	chr11:533266-533652	chr11:533734-533974	chr11:534176-534326
Exon length (bp)	241	387	241	151
GC%	67.63	66.93	61.41	60.93
Mean depth of coverage	4.56X	3.96X	7.68X	8.00X
% covered with at least 1X depth	99.59	100	100	100
% covered with at least 5X depth	55.6	42.12	82.57	100
% covered with at least 10X depth	0	0	36.51	5.96
% covered with at least 20X depth	0	0	0	0
% covered with at least 30X depth	0	0	0	0

Table S5 : Output of Mapinsights *siteinfo* module on a missense polymorphic variant site (chr13:32906729-32906729) of *BRCA2* gene in a 1000G project dataset (HG01402 sample). Five reads are aligned in this location of the genome. 'A' is the reference base in this coordinate but all aligned reads contain base 'C' indicating it's an "other allele homozygous" site. Quality of most of the C bases are good except one where the base quality is 9. Four out of five reads are second in pair reads. Other relevant information such as strand, read positions, mapping quality, insert-size etc about the site can be obtained from the table.

For each input site, there are two blocks in the output. The first output block provides information like genomic coordinates, reference-base, 20bp flanks with GC% and depth. Depending on the depth the second block provides following information as listed
1st column : Aligned bases at input position
2nd column : Quality value of aligned bases at input position
3rd column : Strand
4th column : First in pair [rd1] / Second in pair [rd2]
5th column : Position in read
6th column : Proper pair [pp] / Not proper pair [npp]
7th column : RG-tag @RG:ID
8th column : MD-tag
9th column : Number of CIGAR operation
10th column : Mapping quality
11th column : Insertsize
12th column : Read-Id

chr13:32906729-32906729 ref_base = A AAATGTAGCA A ATCAGAAGCC GC% [in 20bp flanks] = 40.00 depth = 5

C	30	-	rd2	88	pp	HG01402_FCD1LM1ACXX_L1	87A12	1	60	452	ERR251180.55313504
C	28	+	rd2	61	pp	HG01402_FCD1LM1ACXX_L1	60A39	1	60	462	ERR251180.10393056
C	30	+	rd2	44	pp	HG01402_FCD1LM1ACXX_L1	43A56	1	60	469	ERR251180.65551447
C	9	+	rd2	7	pp	HG01402_FCD1LM1ACXX_L1	6A92C0	1	60	464	ERR251180.51028629
C	28	+	rd1	4	pp	HG01402_FCD1LM1ACXX_L1	3A96	1	60	440	ERR251180.33450544

Table S6 : Logs generated by Mapinsights *jumpreads* module in a 1000G project dataset (HG01402 sample)

Mapping properties	No of reads
Mapped-pair forward	1070254
Mapped-pair reverse	1074636
Mapped-pair outward	500954
Insertsize>=1.00K	1635014
Mapped-to-diffChr	2503542

Table S7 : Descriptive statistics of substitution rate across sequencing platforms

	All cycles					
	NovaSeq (NYGC)	NovaSeq (NIBMG)	HiSeq (PLPED)	HiSeq (GIAB)	MGI	AVITI
Average	1.37	1.30	1.58	1.50	1.15	1.53
Median	1.26	1.22	1.54	1.51	0.87	1.36
Max	2.29	2.14	4.74	2.74	3.85	3.10
Min	0.80	0.80	1.06	1.01	0.56	0.71

	Last 25 cycles					
	NovaSeq (NYGC)	NovaSeq (NIBMG)	HiSeq (PLPED)	HiSeq (GIAB)	MGI	AVITI
Average	2.07	1.92	2.10	1.76	2.46	2.35
Median	2.09	1.93	2.03	1.72	2.41	2.32
Max	2.29	2.14	4.74	2.74	3.85	3.10
Min	1.87	1.75	1.74	1.52	0.62	1.89

Table S8 : Download source of NA12878 CRAM and VCF

Sample	Source	Type	LINK
NA12878	GIAB	Benchmarked VCF	https://ftp-trace.ncbi.nlm.nih.gov/ReferenceSamples/giab/release/NA12878_HG001/latest/GRCh38/
	NYGC	VCF	http://ftp.1000genomes.ebi.ac.uk/vol1/ftp/data_collections/1000G_2504_high_coverage/working/20201028_3202_raw_GT_with_annot/
	NYGC	cram	http://ftp.sra.ebi.ac.uk/vol1/run/ERR323/ERR3239334/NA12878.final.cram

Table S9 : List of predictor variables, logs and results of logistic regression analysis

		Odds Ratios	
Variable_name	Description	Heterozygous Class-true	Other allele homozygous Class-true
highmapq1mm	Mutant reads: Mapping quality >=50 with 1 mismatch	1.0677	1.0282
highmapq2mm	Mutant reads: Mapping quality >=50 with 2 mismatch	1.062	1.0199
highmapq3mm	Mutant reads: Mapping quality >=50 with 3 mismatch	1.0439	1.0106
highmapq4amm	Mutant reads: Mapping quality >=50 with >=4 mismatch	0.9804	0.991
lowmapq1mm	Mutant reads: 0 < Mapping quality < 50 with 1 mismatch	0.9442	0.939
lowmapq2mm	Mutant reads: 0 < Mapping quality < 50 with 2 mismatch	0.9477	0.9508
lowmapq3mm	Mutant reads: 0 < Mapping quality < 50 with 3 mismatch	0.9381	0.9685
lowmapq4amm	Mutant reads: 0 < Mapping quality < 50 with >=4 mismatch	0.8103	0.9377
zeromapq1mm	Mutant reads: Mapping quality == 0 with 1 mismatch	0.6828	0.7819
zeromapq2mm	Mutant reads: Mapping quality == 0 with 2 mismatch	0.6622	0.8596
zeromapq3mm	Mutant reads: Mapping quality == 0 with 3 mismatch	0.4666	0.789
zeromapq4amm	Mutant reads: Mapping quality == 0 with >=4 mismatch	0.4699	0.6778
isizezero	Mutant reads: Insert-size = 0	0.6698	0.7874
isize1k	Mutant reads: Insert-size >= 1K	0.4853	0.6113
isizenorm	Mutant reads: 0 < Insert-size < 1K	0.9838	1.0227
clipindel	Mutant reads: Clipping and indel counts	0.9307	0.9552
refpoly	Reference base homopolymer	0.863	0.9439
varpoly	Variant base homopolymer	0.8715	0.9354
wildmismtch	Non-mutant reads : mismatch counts	0.9638	0.847
wildzeromapq	Non-mutant reads : mapping quality == 0	0.9436	1.027
wildlowmapq	Non-mutant reads : 0 < mapping quality < 50	0.9101	1.1429
wildhighmapq	Non-mutant reads : mapping quality >= 50	0.9858	0.8095
wildisizezero	Non-mutant reads: Insert-size = 0	0.9587	1.0983
wildisize1k	Non-mutant reads: Insert-size >= 1K	0.6634	0.8546
wildisizenorm	Non-mutant reads: 0 < Insert-size < 1K	0.9778	0.9612
wildclipindel	Non-mutant reads: Clipping and indel counts	0.9244	0.7506

**Heterozygous**			
		Predicted true	Predicted false
Actual true	12900	11784	1116
Actual false	12900	3649	9251
Accuracy		81.53%	

**Other allele homozygous**			
		Predicted true	Predicted false
Actual true	6300	5354	946
Actual false	6300	2496	3804
Accuracy		72.68%	

Table S10 : Exon-wise depth status of *CDKN2A* gene in sequence data generated using Nextera and Truseq library preparation kits

No of exones	4	
Target bed size (bp)	1790	
Mean depth of coverage	19.38X (Nextera)	24.55X (TruSeq)

Exon no	Exon1		Exon2		Exon3		Exon4	
Genomic coordinates	chr9:21967751-21968241		chr9:21970900-21971207		chr9:21974402-21975038		chr9:21994137-21994490	
Exon length (bp)	491		308		637		354	
GC%	43.38		72.4		64.99		71.19	
Library	Nextera	TruSeq	Nextera	TruSeq	Nextera	TruSeq	Nextera	TruSeq
Mean depth of coverage	19.36X	14.49X	12.50X	17.12X	22.16X	39.43X	20.40X	18.22X
% covered with at least 1X depth	99.8	74.54	86.36	100	99.84	81.95	99.72	85.03
% covered with at least 5X depth	99.39	61.91	57.47	100	99.69	63.27	88.7	60.45
% covered with at least 10X depth	77.6	36.25	42.21	98.7	82.42	58.24	71.75	51.98
% covered with at least 20X depth	41.34	25.87	31.82	27.92	59.34	49.45	51.69	32.2
% covered with at least 30X depth	20.77	16.29	19.81	0	25.27	42.07	29.38	24.86
